# Natural Products against Human and Veterinary Trichomonads: From In Vitro Activity to Future Therapeutic Perspectives

**DOI:** 10.1021/acsomega.6c08481

**Published:** 2026-10-19

**Authors:** Bárbara B. R. Soares, Saulo A. Menezes, Tiana Tasca

**Affiliations:** Grupo de Pesquisa em Tricomonas, Programa de Pós-Graduação em Ciências Farmacêuticas, Faculdade de Farmácia e Centro de Biotecnologia, 28124 Universidade Federal do Rio Grande do Sul, 90610-000 Porto Alegre, RS, Brazil

## Abstract

Pathogenic trichomonads are flagellated protozoan parasites of the urogenital and digestive tracts of humans and animals. These organisms impose substantial health and economic burdens on the world. *Trichomonas vaginalis* causes human urogenital trichomoniasis, the most common nonviral sexually transmitted infection worldwide, and has been associated with adverse reproductive outcomes and increased risk of HIV transmission. In animals, *Trichomonas gallinae* causes avian trichomoniasis, a disease of the upper digestive tract that may impair feeding, compromise survival, and contribute to bird population declines, whereas *Tritrichomonas foetus* is responsible for bovine trichomoniasis, a major cause of cattle production losses. Despite their importance, therapeutic options remain limited. Human and avian trichomoniasis still rely on 5-nitroimidazoles, but drug resistance and regulatory restrictions have become important concerns, whereas no approved pharmacological treatment is currently available for bovine trichomoniasis, and infected animals are culled. In this context, natural products have emerged as valuable sources of new antitrichomonal agents. This Review summarizes 84 studies published between 2016 and 2026 investigating natural products against *T. vaginalis*, *T. gallinae*, and *T. foetus*, including plant-derived extracts and fractions, essential oils, isolated compounds, microbial metabolites, and nanotechnology-based formulations. Although several products displayed relevant antitrichomonal activity, most evidence remains restricted to *in vitro* studies, with comparatively few *in vivo* evaluations and no clinical validation of natural-product-derived candidates for trichomoniasis. Overall, natural products represent a chemically diverse and still underexplored source of antitrichomonal leads, but further validation of their selectivity, pharmacological properties, and *in vivo* efficacy is required before their therapeutic potential can be established.

## Graphical abstract

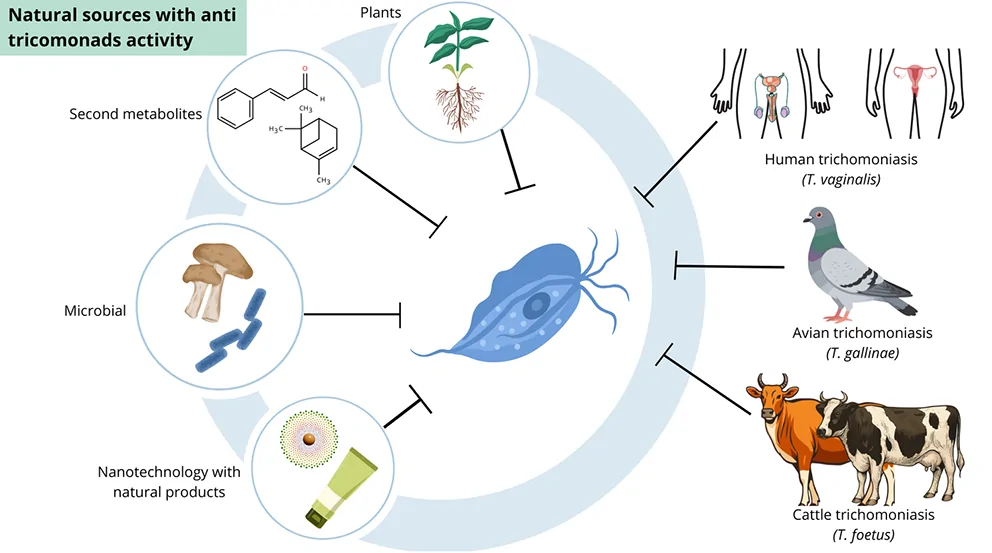

## 1 Introduction

*Trichomonas vaginalis* is an extracellular protozoan parasite and the etiologic agent of human trichomoniasis, the most common nonviral sexually transmitted infection (STI) worldwide. According to the World Health Organization (WHO), human trichomoniasis has an estimated incidence of approximately 156 million new cases annually, a figure higher than that of other curable STIs, such as chlamydia, gonorrhea, and syphilis.^1^ As the parasite infects the squamous epithelial cells of the human urogenital tract, symptomatic women may present with vaginal discharge with a yellow/greenish color and unpleasant odor, dysuria, vaginitis, and a cervix with hemorrhagic spots, known as “*colpitis macularis*.” In men, urethritis and prostatitis are the most common symptoms.^2,3^ About 80% of trichomoniasis cases are asymptomatic in both men and women.^4^ This scenario facilitates the persistent contact of *T. vaginalis* with host cells, which can lead to severe consequences, including infertility, pelvic inflammatory disease, premature birth/pregnancy disorders, prostate and cervical cancer, and acquisition and transmission of HIV.^5,6^

Human trichomoniasis is a curable infection; however, it is not notifiable, and there is a high number of asymptomatic cases. Sexual practices with multiple partners, bacterial vaginosis, poor urogenital hygiene, and ineffective sexual health policies are some factors linked to an increased risk of infection.^7^ The increased rate of *T. vaginalis* infection in some regions may be related to the lack of sensitive diagnostic methods, effective treatment, and ineffective sex education, such as condom use. However, even though condom use is the most effective method of STI prevention, its use is restricted in some countries due to religious beliefs or cultural customs.^8^ The most common, but relatively insensitive, way to diagnose trichomoniasis is the wet mount through direct microscopic examination of vaginal or urethral secretions or urine samples, which makes it possible to observe active and motile trichomonads. This technique offers rapid results, allowing immediate treatment, but it depends on fresh samples and the expertise of the microscopist to note the trophozoites.^9,10^

Currently, only the 5-nitroimidazole drugs are approved by the Food and Drug Administration (FDA) for the treatment of human trichomoniasis: metronidazole (MTZ; first-line treatment), tinidazole (TNZ), and secnidazole (SCZ) (Figure 1). SCZ was approved by the FDA in 2017 for the treatment of bacterial vaginosis and, subsequently, in 2021, for trichomoniasis.^11^ These prodrugs penetrate parasite membranes by simple diffusion and reach the hydrogenosome. In this compartment, pyruvate metabolism drives electron flow through pyruvate:ferredoxin oxidoreductase (TvPFOR) and ferredoxin (Fd). Because nitroimidazoles have a high affinity for this low-redox electron transfer system, they can compete for electrons and function as preferred electron acceptors. This promotes the reduction of the nitro group, leading to the formation of cytotoxic intermediates that damage DNA, RNA, proteins, and lipids, ultimately killing the parasite.^12^ The treatment of trichomoniasis presents some problems: to be effective, both sexual partners must be treated and use condoms until the end of therapy to avoid reinfection by the parasite. Additionally, available medications can cause adverse effects, such as hypersensitivity, headache, cutaneous rash, hives, angioedema, fever, and gastrointestinal distress.^2^ These adverse effects lead to premature discontinuation of treatment, which increases the transmission rate and contributes to *T. vaginalis* resistance to 5-nitroimidazole drugs.^13^

**Figure 1:**
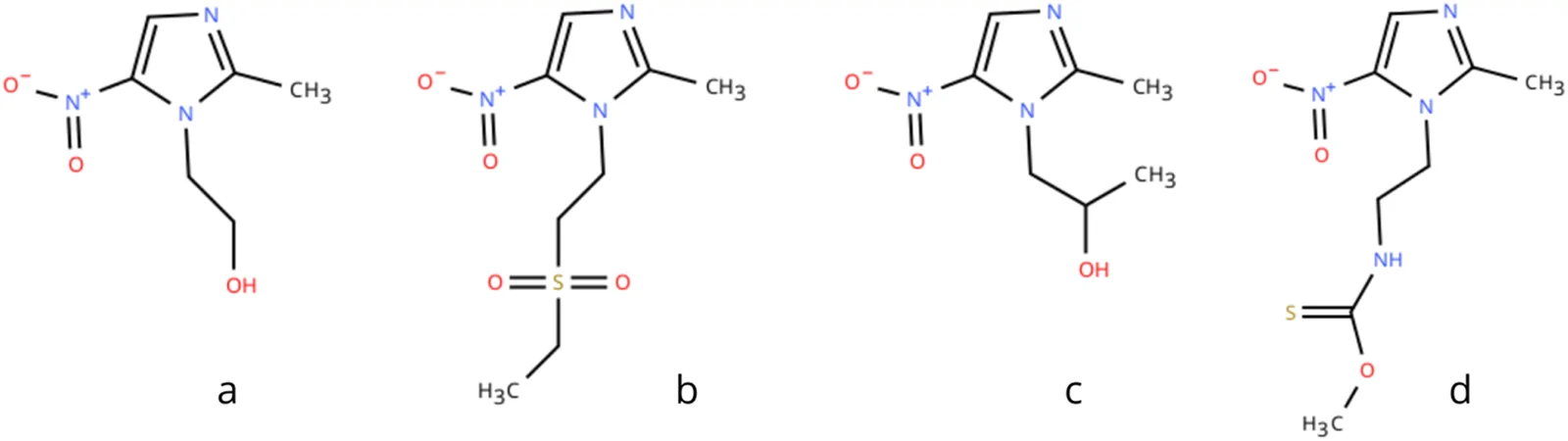
Chemical structures of nitroimidazoles used for human and veterinary trichomoniasis: MTZ (a), TNZ (b), SCZ (c), and carnidazole (d).

The most worrying part of this scenario is that there is no drug approved to replace 5-nitroimidazoles, and in cases of resistance, only drug dose management is performed.^2,14^ In clinical routine, at least 10% of cases are resistant, and cross-resistance between 5-nitroimidazoles is common.^7,15^ Resistance to 5-nitroimidazoles can occur in two different forms: aerobic and anaerobic. The aerobic type is associated with high intracellular oxygen concentration, which makes drug activation difficult because oxygen competes with 5-nitroimidazole for electrons. Anaerobic resistance is associated with continuous *in vitro* cultivation, in which *T. vaginalis* is cultured with increasing concentrations of MTZ.^12^ Unfortunately, the prevalence of resistant isolates is not controlled. Some studies show that there is an increase in the prevalence of resistant *T. vaginalis* between 2.5 and 9.6%.^16,17^ A study revealed *T. vaginalis* isolates from KwaZulu-Natal (South Africa) with resistance levels of 11, 2, and 1% for MTZ, TNZ, and SCZ, respectively; and the intermediate resistance scores were 28% for MTZ, 18% for TNZ, and 24% for SCZ.^18^ In fact, the true proportion is unknown and underestimated, as MTZ susceptibility testing of *T. vaginalis* isolates is performed only in specialized laboratories mainly for research purposes. Currently, there are no options for the oral treatment of trichomoniasis other than the drugs already mentioned. This difficult scenario sheds light on the importance of studying new alternatives to improve the management of human trichomoniasis.

*Trichomonas gallinae* is a microaerophilic flagellated protozoan and the causative agent of avian trichomoniasis in domestic and wild birds, especially Columbiformes, Falconiformes, and Strigiformes. The infection in pigeons is commonly termed “canker,” and the rock pigeon (*Columba livia*) is considered the primary host and an important driver of the parasite’s wide geographic distribution.^19,20^ Transmission is strongly linked to close contact behaviors, including communal feeding and drinking stations, feeding of chicks by infected parent birds through crop milk (a nutrient-rich secretion), and consumption of infected birds by predatory birds.^21^ Although *T. gallinae* possesses only the trophozoite stage in its life cycle, cyst-like stages (pseudocysts) have been reported as potentially enhancers of survival outside the host, which could facilitate transmission under unfavorable conditions.^19,22,23^

Avian trichomoniasis is primarily a disease of the upper digestive tract characterized by proliferative lesions that can compromise feeding and, in severe cases, become fatal. Affected birds may show nonspecific symptoms such as lethargy, reduced flight activity, weight loss, increased water intake, head shaking, dysphagia, regurgitation, nasal discharge, dyspnea, and diarrhea.^24^ Lesion severity varies widely, ranging from mild mucosal inflammation to extensive caseous masses that can obstruct the esophageal lumen, impairing nutrient intake and causing death by asphyxiation. In highly virulent infections, invasion beyond the primary site may occur, including extension into the head (sinuses, brain, orbital regions) and systemic dissemination with involvement of the liver and other viscera.^25^ It is of great concern that *T. gallinae* infection outbreaks can decrease bird populations and alter community structure, which indirectly affects ecosystem processes supported by birds, such as pest control, pollination, and seed dispersal. In addition, the parasite is not only a wildlife concern; its circulation in domestic bird settings increases sanitary risk at the wildlife-domestic interface and may generate commercial repercussions for the poultry sector, including constraints on movement and trade and associated economic losses.^26^

Nitroimidazoles, such as MTZ, dimetridazole, ronidazole, and carnidazole (Figure 1), have been considered the standard treatment for avian trichomoniasis, with MTZ being widely used in clinical practice.^27^ However, resistance to these drugs was first noted for racing pigeons and is already documented in *T. gallinae*.^28–30^ In addition, the carcinogenicity of MTZ has led to the prohibition of its use in food-producing animals in many countries, including the pigeon industry.^31,32^ From this perspective, the search for treatment alternatives is of utmost importance.

*T. foetus* is a flagellated protozoan and the causative agent of bovine trichomoniasis. Its anatomy includes four flagella, with three anterior flagella and one posterior flagellum, in addition to a single undulating membrane.^33^ The transmission of trophozoites occurs during the course of sexual activity, thereby contributing to a decline in productivity in systems dedicated to the raising of cattle.^34^ Furthermore, domestic cats (*F. catus*) have been observed to become infected with *T. foetus*, a parasite that is transmitted via the fecal-oral route and causes diarrhea.^35^ The trophozoites infect the genitourinary system of bulls, which are considered asymptomatic and potentially permanent carriers and transmitters of the infection.^36^ In bovines, the infection leads to a range of reproductive health complications, including pyometra, reduced birth rates, pregnancy loss, endometritis, vaginitis, decreased calving ratios, and infertility.^34,37–39^ The diagnosis of trophozoites is performed through the analysis of smegma samples obtained from bulls or vaginal wash samples collected from cows. *T. foetus* is isolated through culture and microscopic analysis, with the use of polymerase chain reaction (PCR) providing confirmatory evidence.^40^

Bovine trichomoniasis, in addition to its role in causing reproductive failures in cattle, has also been associated with significant financial losses due to reduced calf and milk production. This is due to the parasite’s ability to persist in both raw and processed semen of bulls, facilitating the transmission of the disease during artificial insemination.^41^ The financial impact of *T. foetus* can reach approximately 1 billion dollars in the USA.^34,42,43^ Between 2015 and 2019, bovine trichomoniasis was identified in 25 states in the United States, with a total of 3,817 cases reported nationwide.^34^ In Brazil, *T. foetus* has been detected in the states of Rio Grande do Sul,^44^ Alagoas,^45^ Paraíba,^46^ Pernambuco,^47^ and Minas Gerais.^48^ These data are concerning, given that there is no effective treatment or vaccine, and natural mating is still the predominant reproductive method (80%).^49^ Notably, the FDA prohibits the use of nitroimidazoles in food-producing animals.^50^ The TrichGuard vaccine necessitates two annual booster doses and does not fully prevent infection in vaccinated cattle. It does not induce long-term immunological memory or prevent abortion, although it stimulates the production of anti-*T. foetus* antibodies and increases conception rates.^38,51,52^ Consequently, infected bulls are not treated but are instead sent for slaughter. Considering the prevailing constraints, there is an imperative for the expeditious formulation of novel therapeutic and preventive methodologies that demonstrate enhanced efficacy in the management of bovine trichomoniasis. The quest for solutions is imperative to mitigate the adverse effects of this disease on herd reproductive health and to enhance the efficiency of livestock production.

### 1.1 Potential of Natural Products as Sources of New Trichomonacidal Molecules

Considering the neglect of human and animal trichomoniasis, there is an urgent need to develop novel, affordable, and safe drugs to treat these infections. In this context, interest in natural products, particularly those derived from plants, as sources of lead molecules has increased considerably in recent years. It is estimated that the global number of medicinal plant species is approximately 250,000.^53,54^ However, only 6% of these have known biological properties and 0.75% of medicinal plant compounds have been scientifically studied. Notably, the efficacy of most of these compounds is limited to *in vitro* studies, with only a limited number of them advancing to the preclinical stage.^55^ Despite this, natural products have been demonstrated to be significant sources of metabolites, and their use as active principles in the treatment of numerous diseases has been well documented.^56^

The significance of natural products as novel therapeutic agents stems from their inherent diversity, which is predominantly derived from renewable resources. These compounds exhibit characteristics such as chirality, optical activity, stereochemistry, and regioselectivity, which are crucial for the development of effective pharmaceuticals to combat diseases.^57^ Natural products have evolved to perform specific biological functions and play key roles in regulating endogenous defense mechanisms and interacting, often competitively, with other organisms. This underscores their significant relevance to infectious diseases.^58^

Plant-derived natural products represent valuable starting points for drug discovery because of their broad chemical diversity and potential accessibility.^59,60^ From 1981 to 2019, 41.9% of the 1,881 approved drugs were associated with natural products or natural-product-derived scaffolds. Among the 20 antiparasitic agents approved during this period, nine were natural products or natural-product-derived compounds.^61,62^ Several researchers have noted the potential of plants to act as antitrichomonad agents. In this regard, plant screening initiatives have identified that several species used in folk medicine exhibit trichomonacidal activity.^63,64^

There are currently numerous molecules under investigation in clinical trials (ClinicalTrials.gov) for the treatment of human trichomoniasis, including both novel compounds and already approved drugs being evaluated in alternative dosing regimens or routes of administration.^65^ Across different therapeutic areas, 125 natural products or natural-product-derived compounds have been reported as undergoing clinical trials or registration processes.^66^ However, to date, we have identified no clinical trials involving natural-product-derived compounds specifically for trichomoniasis.

Importantly, a distinction should be made between bioactive natural products and potential drugs. In this Review, natural products exhibiting antitrichomonal activity in screening studies are considered as bioactive compounds; however, biological activity alone does not qualify them as drug candidates. Progression toward drug candidacy requires additional evidence regarding selectivity, toxicity, mechanism of action, efficacy in relevant *in vivo* models, pharmacokinetic properties, and overall pharmacological suitability. Thus, the chemical diversity of natural products supports their continued exploration as sources of trichomonacidal scaffolds. In this context, distinctive cellular structures and organelles of trichomonads may also provide relevant opportunities for therapeutic targeting (Figure 2).

**Figure 2:**
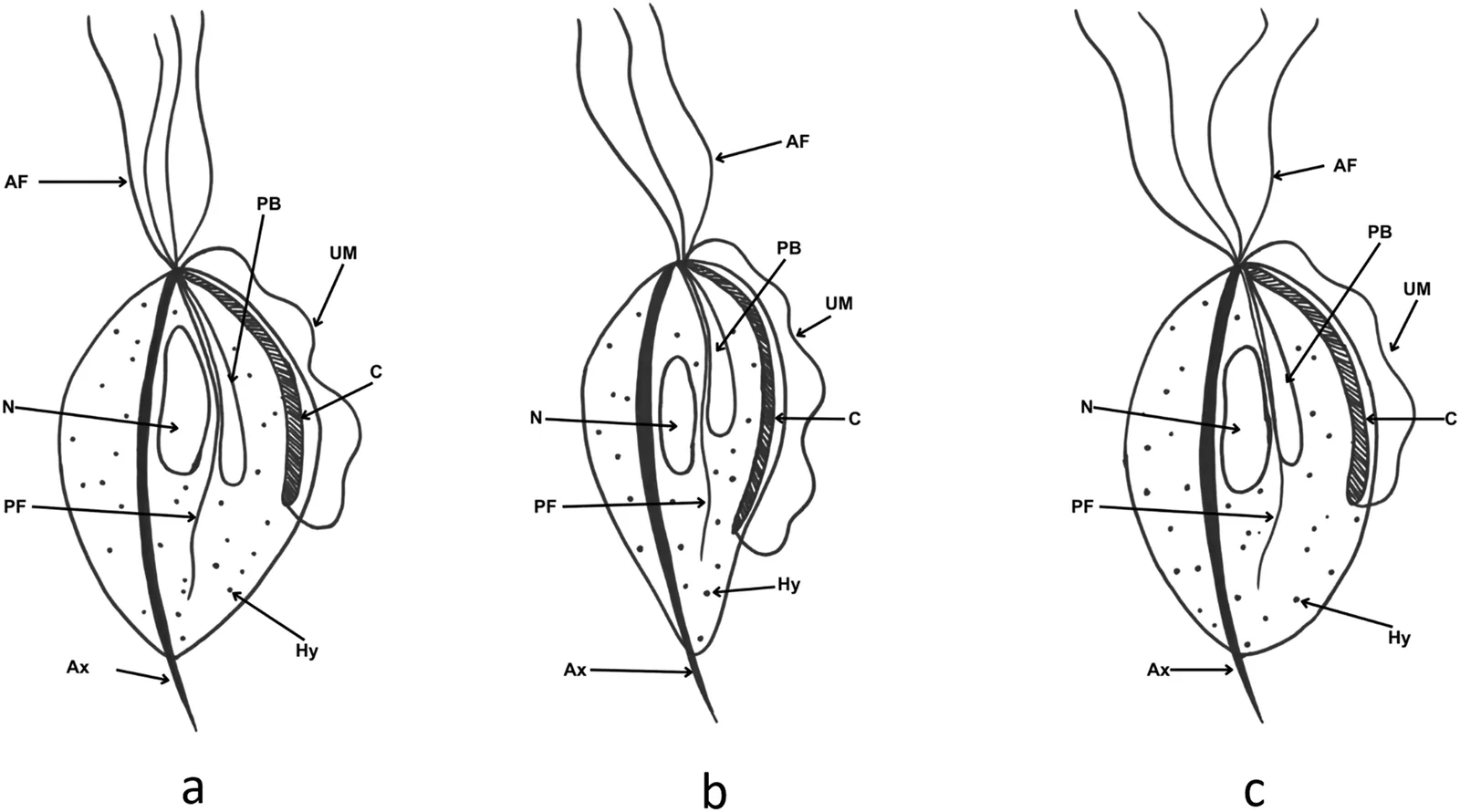
Schematic representation of the main morphological structures of trichomonads: (a) *Trichomonas vaginalis*; (b) *Tritrichomonas foetus*; and (c) *Trichomonas gallinae*. Cellular structures: AF: anterior flagella, involved in parasite locomotion; PB: parabasal body, a Golgi-associated structure connected to the cytoskeleton and involved in cellular organization and secretion; UM: undulating membrane, a membrane specialization associated with the recurrent flagellum that contributes to parasite motility; C: costa, a striated cytoskeletal structure that supports the undulating membrane; N: nucleus, containing the trophozoite’s genetic material; PF: pelta–axostylar complex-associated filament, a cytoskeletal structure contributing to cell organization and morphology; HY: hydrogenosomes, mitochondrion-related organelles involved in anaerobic energy metabolism, ATP production, and 5-nitroimidazole activation; AX: axostyle, a microtubule-based cytoskeletal structure that provides structural support, contributes to cell shape, and extends posteriorly from the cell.

Despite increasing interest in natural products with antitrichomonal activity, the available evidence remains fragmented, and an integrated assessment comparing human and veterinary trichomonads is still lacking. Therefore, this review discusses the available literature published between January 2016 and January 2026 on the antitrichomonal activity of natural products against relevant pathogenic trichomonads, including *T. vaginalis*, *T. gallinae*, and *T. foetus*. A total of 303 papers were retrieved from this period, comprising *in vitro*, *in vivo*, and *in silico* studies. The available information published in English or Spanish was gathered using PubMed/Medline, Web of Science, and Scopus using the following search strategy: (“natural product*” or “Tritrichomonas foetus” or “Trichomonas gallinae” or “trichomoniasis” or “trichomonacidal” or “anti-Trichomonas”). Articles that did not specifically address the activity of natural products against the above-mentioned trichomonads, studies for which full-text access was not available, reviews on natural products, and articles that were duplicates were excluded from analysis.

The narrative review was organized according to the origin and type of natural product, including plant-derived extracts and fractions, essential oils, and other plant products, isolated compounds, microbial natural products, and formulation-based approaches. Within each category, studies were discussed primarily according to the trichomonad species evaluated, following the order *T. vaginalis*, *T. gallinae*, and *T. foetus*, while emphasizing the most biologically and pharmacologically relevant findings.

For comparison, MTZ control data, including half-maximal inhibitory concentration (IC_50_), half-maximal effective concentration (EC_50_), minimum inhibitory concentration (MIC), and minimum lethal concentration (MLC) values, are provided in the tables whenever reported in the original study; when absent, the corresponding control data were not reported by the original authors. Also, crude extracts, essential oils, and fractions showing antitrichomonal activity at concentrations ≤ 100 μg/mL were classified as highly active, whereas isolated compounds were classified as highly active when activity was reported at concentrations ≤ 10 μM or ≤ 5 μg/mL, based on IC_50_, EC_50_, MIC, and MLC values. The selectivity index (SI), when available, was also considered in the overall assessment of biological relevance and therapeutic potential. Accordingly, the term “promising” was reserved for products that combined relevant antitrichomonal activity with additional favorable evidence, particularly parasite selectivity or activity against drug-resistant isolates.

Given the substantial methodological heterogeneity among the included studies, numerical values and concentration units were generally retained as reported in the original studies to preserve the original data and avoid introducing artificial uniformity or a potential misinterpretation. To the best of our knowledge, this is the first comprehensive review dedicated to assessing the potential of natural products against human and veterinary trichomonads.

## 2 Results and Discussion

### 2.1 Natural Products from Plants with Trichomonacidal Activity

After application of the eligibility criteria, 84 studies were included for data extraction. Across these publications, we compiled 69 plant species distributed among 32 botanical families (Table 1). The most represented families were Lamiaceae (14 species), followed by Myrtaceae (5 species), Asteraceae (4 species), and Zingiberaceae (4 species); additional families with multiple representatives included Apiaceae, Fabaceae, Poaceae, and Rutaceae (3 species each). Most studies using plant extracts, fractions, or essential oils were conducted *in vitro* (89.29%), whereas 6 studies (10.71%) combined *in vitro* and *in vivo* analyses. Regarding the trichomonad species evaluated, 43 studies (76.79%) investigated *T. vaginalis*, 11 (19.64%) investigated *T. gallinae*, and 5 (8.93%) investigated *T. foetus*. A small subset of publications (3 studies; 5.36%) assessed more than one trichomonad species (all of them tested *T. vaginalis* + *T. foetus* in the same study) (Table 1).

**Table 1:** Natural Products from Plants with Trichomonacidal Activity Reported against Human and Veterinary Trichomonads^a^

botanical family/plant species	plant part used	type of product	trichomonad (isolate)	active concentration	study type	chemical composition	toxicity studies	ref
**Amaryllidaceae**								
*Allium sativum* L.	Bulbs	AEX	*T. vaginalis*	30% of AEX inhibited parasites completely (24 h)	*In vitro*	N.d.	N.d.	^73^
**Anacardiaceae**								
*Pistacia khinjuk* Stocks	Fruit	MEX	*T. vaginalis*	IC_50_ = 93.6 μg/mL (24 h) and 26.6 μg/mL (48 h)	*In vitro*	N.d.	N.d.	^72^
				MTZ (control)_IC50_ = 0.5 μg/mL (24 h) and 0.09 μg/mL (48 h)				
**Apiaceae**								
*Cnidium monnieri* (L.) Cusson	Fruit	EtEX	*T. vaginalis*	At 12.5 mg/mL, inhibited the parasite’s growth	*In vitro*	Osthole, xanthotoxol, bergapten, and isopimpinellin	N.d.	^74^
*Foeniculum vulgare* Mill.	Seed	HEX, MEX, AEX	*T. vaginalis* (Tv3, 15, 16, 17, and 18)	HEX and MEX _MLC_ = 400 μg/mL (24 h) and 360 μg/mL (48 h)	*In vitro*	N.d.	N.d.	^75^
				MTZ (control)_MLC_ = 13.7 μg/mL (24 h) and 6.8 μg/mL (48 h)				
	Seed	EO	*T. vaginalis* (Tv3, 15, 16, 17, and 18)	MLC = 1600 μg/mL	*In vitro*	E-anethole (88.41%)	N.d.	^75^
				MTZ (control)_MLC_ = 13.7 μg/mL (24 h) and 6.8 μg/mL (48 h)				
*Trachyspermum ammi* (L.) Sprague	N.r.	EO	*T. vaginalis*	IC_50_ = 69.47 μg/mL (48 h)	*In vitro*	Thymol (47.05%), γ-terpinene (26.78%), and p-cymene (22.06%)	N.d.	^76^
**Apocynaceae**								
*Asclepias curassavica* L.	Leaves, stem	EtEX	*T. vaginalis*	IC_50_ = 302 μg/mL	*In vitro/In vivo*	Palmitic acid	Primary murine macrophages’ viability was not affected; Symptoms of toxicity were not observed in mice (1000 mg/kg)	^77^
*Caralluma procumbens* Gravely & Mayur.	Stem	MEX	*T. foetus* (ATCC50152)	10 mg/mL of MEX inhibited parasites completely (48 h)	*In vitro*	Phenols, tannins, alkaloids, and glycosides	N.d.	^71^
**Asteraceae**								
*Artemisia aucheri* Boiss.	Leaves	HAEX	*T. vaginalis*	IC_50_ = 144.5 μg/mL	*In vitro*	N.d.	N.d.	^78^
				MTZ (control)_MLC_ = 16.0 μg/mL				
*Artemisia ludoviciana* Nutt.	Whole plant	EO	*T. vaginalis*	MLC = 4.0 mg/mL	*In vitro*	Camphor (40.57%) and 1,8-cineole (25.53%)	N.d.	^79^
*Artemisia sieberi* Besser ★	Aerial parts	EO	*T. gallinae*	MIC = 10 μg/mL (24 h); MTZ (control)_MIC_ = 20 μg/mL (24 h).	*In vitro/In vivo*	α-thujone (31.5%), β-thujone (11.92%), camphor (12.3%), and 1,8-cineole (10.09%)	No mortality and no clinical side effects were observed in	^68^
				The EO (25–50 mg/kg) significantly reduced the number of *T. gallinae* recovered from pigeons and led to full recovery on day 5 p.i.			treated birds with EO at 25 and 50 mg/kg	
*Pulicaria dysenterica* (L.) Bernh.	Aerial parts	MEX	*T. gallinae*	MIC = 6.25 mg/mL (6 h)	*In vitro*	N.d.	N.d.	^80^
**Berberidaceae**								
*Berberis vulgaris* L.	Root	MEX	*T. vaginalis* (GT15)	IC_50_ = 192.08 μg/mL	*In vitro*	Alkaloids	Low level of hemolysis (<7%) up to 100 μg/mL	^81^
				MTZ (control)_IC50_ = 0.07 μg/mL				
**Boraginaceae**								
*Echium plantagineum* L.	Flowers, leaves	EO, MEX, HEX, EAEX, EtEX, IFEX	*T. gallinae*	EtEX (leaves) led to 95% parasite mortality at 0.4 mg/mL; IFEX (flowers) reduced parasite viability in 94.6% at the same concentration	*In vitro*	EtEX (leaves): echimidine (5.8%) and echiumine-NOx (1.87%); IFEX (flowers): echimidine (4.93%), intermedine (2.62%), heliotrine-NOx (1.72%), and echiumine-NOx (1.18%)	N.d.	^82^
**Caryocaraceae**								
*Caryocar coriaceum* Wittm.	Inner mesocarp	MEX	*T. vaginalis* (ATCC 30236)	IC_50_ = 662 μg/mL	*In vitro*	N.d.	MEX at 1,000 μg/mL showed low fibroblast mortality (<15%); No toxicity was observed in the *D. melanogaster* model at 0.5 mg/g	^83^
**Celastraceae**								
*Elaeodendron trichotomum* Thunb.	Leaves, stem, root	DEX	*T. vaginalis* (GT3)	IC_50_ (root) = 0.46 μg/mL	*In vitro*	Tingenone (3.84%) and pristimerin (0.14%)	N.d.	^67^
				MTZ (control)_IC50_ = 0.12 μg/mL				
**Crassulaceae**								
*Kalanchoe daigremontiana* (Raym.-Hamet & Perrier) A.Berger	Leaves	MEX	*T. vaginalis* (GT15)	IC_50_ = 105.27 μg/mL	*In vitro*	N.d.	Low level of hemolysis (<8%) up to 1000 μg/mL	^84^
				MTZ (control)_IC50_ = 0.17 μg/mL				
**Cupressaceae**								
*Juniperus communis* L.	Fruit	EtEX, MEX, CHEX, AEX	*T. vaginalis* (ATCC50143)	EtEX, MEX, AEX _IC50_ > 250 μg/mL, CHEX _IC50_ = 30 μg/mL	*In vitro*	N.d.	N.d.	^85^
**Fabaceae**								
*Ceratonia siliqua* L.	Pods	AEX	*T. gallinae*	MIC = 50 μg/mL (24 h)	*In vitro*	Nonadecane (23.34%)	N.d.	^86^
				MTZ (control)_MIC_ = 50.0 μg/mL				
*Dalbergia ecastophyllum* (L.) Taub.	Resinous exudate (Brazilian red propolis)	EO	*T. vaginalis* (ATCC 30236)	MIC = 500 μg/mL,	*In vitro*	Methyl eugenol (13.1%), (E)-β-farnesene (2.5%), and δ-amorphene (2.3%)	No cytotoxicity up to 250 μg/mL against CHO-K1 cells	^87^
				IC_50_ = 100 μg/mL				
*Poincianella microphylla* (Mart. ex G.Don) L.P.Queiroz	Fruit	AEX and fractions	*T. vaginalis* (ATCC 30236)	M5 fraction IC_50_ = 70.41 μg/mL, M10 fraction IC_50_ = 142.1 μg/mL	*In vitro/In vivo*	O-digalloyl hexoside and O-(digalloyl) quinic acid	M5 and M10 fractions showed no hemolytic activity. Both fractions (500 mg/kg) also caused no significant increase in larvae mortality up to 120 h in the invertebrate model *G. mellonella*	^88^
**Fagaceae**								
*Quercus brantii* Lindl.	Fruits	HAEX	*T. vaginalis*	IC_50_ = 255.1 μg/mL (48 h)	*In vitro*	N.d.	N.d.	^78^
				MTZ (control)_MLC_ = 16.0 μg/mL (24 h)				
*Quercus infectoria* G. Olivier	Bark	MEX	*T. vaginalis*	IC_50_ = 21.3 μg/mL (24 h) and 3.4 μg/mL (48 h)	*In vitro*	N.d.	N.d.	^72^
				MTZ (control)_IC50_ = 0.5 μg/mL (24 h) and 0.09 μg/mL (48 h)				
	Galls	EtEX	*T. vaginalis*	600 μg/mL inhibited 100% of parasitic growth (48 h)	*In vitro*	N.d.	N.d.	^76^
	Galls	EtEX	*T. gallinae*	MIC = 25 μg/mL (24 h)	*In vitro*	β-pinene oxide (21.09%)	N.d.	^86^
				MTZ (control)_MIC_ = 50.0 μg/mL (24 h)				
**Geraniaceae**								
*Pelargonium quercetorum* Agnew	Leaves, flowers, stem	EAEX, HEX, AEX	*T. vaginalis*	EAEX _MIC and IC50_ = 250 and 89 μg/mL, HEX_MIC and IC50_ = 500 and 130 μg/mL, AEX_MIC and IC50_ = 25 and 2.3 mg/mL	*In vitro*	N.d.	N.d.	^89^
				MTZ (control)_MLC_ = 3.1 μg/mL				
*Pelargonium roseum* Willd.	Aerial parts	EO	*T. gallinae*	MIC = 20 μg/mL (24 h); MTZ (control)_MIC_ = 10 μg/mL (24 h). The EO at 50 mg/kg led to a full recovery of infected pigeons on day 6–7 p.i.	*In vitro/In vivo*	N.d.	No mortality or clinical side effects in treated birds (25–50 mg/kg)	^90^
**Lamiaceae**								
*Dracocephalum kotschyi* Boiss	Whole plant	EO	*T. vaginalis*	MLC = 2000 μg/mL (24 h) and 102.8 μg/mL (72 h), IC_50_ = 84.07 μg/mL	*In vitro*	Copaene (20.15%)	CC_50_ = 118.6 μg/mL (24 h); SI = 1.41 (24 h) in J774.A1 cells	^91^
*Lavandula angustifolia* Mill.	Flowering branch	EtEX	*T. gallinae*	MIC = 50 μg/mL (24 h)	*In vitro*	Linalool (26.2%) and borneol (22.7%)	N.d.	^92^
				MTZ (control)_MIC_ = 50.0 μg/mL (24 h)				
*Lavandula luisieri* Riv.-Mart.	Aerial parts	EO	*T. gallinae*	IC_50_ = 103.4 μg/mL	*In vitro*	N.d.	N.d.	^93^
				MTZ (control)_IC50_ = 1.0 μg/mL				
*Mentha arvensis* L.	Leaves	EO	*T. vaginalis*	MLC = 0.11% (24 h) and 0.075% (48 h)	*In vitro*	Menthol (67.2%)	CC_50_ = 0.5% on HeLa cells (SI = 4.5)	^94^
*Origanum majorana* L.	Aerial parts	EO	*T. gallinae*	IC_50_ (hydrodistillation) = 158.8 μg/mL, IC_50_ (steam distillation) = 139.7 μg/mL	*In vitro*	Stem distillation: Thymol (32.7%) and p-cymene (21.0%); Hydrodistillation: 4-terpineol (29.7%)	N.d.	^93^
				MTZ (control)_IC50_ = 1.0 μg/mL				
*Salvia hibrida*	Aerial parts	EO	*T. gallinae*	IC_50_ = 134.6 μg/mL	*In vitro*	N.d.	N.d.	^93^
				MTZ (control)_IC50_ = 1.0 μg/mL				
*Salvia officinalis* L.	Aerial parts	EO	*T. gallinae*	IC_50_ = 139.6 μg/mL	*In vitro*	N.d.	N.d.	^93^
				MTZ (control)_IC50_ = 1.0 μg/mL				
*Salvia sclarea* L.	Aerial parts	EO	*T. gallinae*	IC_50_ = 117.4 μg/mL	*In vitro*	Linalyl acetate (31.2%) and linalool (22.7%)	N.d.	^93^
				MTZ (control)_IC50_ = 1.0 μg/mL				
*Satureja montana* L.	Aerial parts	EO	*T. gallinae*	IC_50_ = 141.4 μg/mL	*In vitro*	N.d.	N.d.	^93^
				MTZ (control)_IC50_ = 1.0 μg/mL				
*Satureja khuzestanica* Jamzad	Leaves	MEX	*T. vaginalis*	IC_50_ = 205.8 μg/mL (24 h) and 5.1 μg/mL (48 h)	*In vitro*	N.d.	N.d.	^72^
				MTZ (control)_IC50_ = 0.5 μg/mL (24 h) and 0.09 μg/mL (48 h)				
	Aerial parts	EO, HEX, MEX, AEX	*T. vaginalis*	HEX _MLC_ = 200 μg/mL, MEX and EO_MLC_ = 400 μg/mL (24 h)	*In vitro*	EO: carvacrol (54.81%), thymol (14.74%), and p-cymene (7.31%)	N.d.	^95^
				MTZ (control)_MLC_ = 12.5 μg/mL (24 h) and 6.2 μg/mL (48 h)				
*Scutellaria havanensis* Jacq.	Aerial parts	MEX, CHEX	*T. vaginalis* (T-H7)	MEX _IC50_ = 20.4 μg/mL, CHEX _IC50_ = 12.9 μg/mL MTZ (control)_IC50_ = 0.62 μg/mL	*In vitro*	CHEX: wogonin (5,7-dihydroxy-8-methoxyflavone) (48.27%); MEX: b-d-Glucopyranose (20.37%)	MEX _CC50_ = 149.6 μg/mL (SI = 7.3) and CHEX _CC50_ = 35.8 μg/mL (SI = 2.8) in peritoneal macrophages	^96^
*Thymus haussknechtii* Velen.	Aerial parts	MEX	*T. vaginalis* (ADUTR011)	MIC = 62.5 μg/mL (48 h)	*In vitro*	N.d.	N.d.	^97^
				MTZ (control)_MIC_ = 15.6 μg/mL				
*Thymus vulgaris* L.	Aerial parts	EO	*T. gallinae*	IC_50_ (hydrodistillation) = 166.0 μg/mL, IC_50_ (steam distillation) = 193.3 μg/mL	*In vitro*	Hydrodistillation: thymol (43.8%) and p-cymene (22.3%); Steam distillation: p-cymene (31.7%) and thymol (28.2%)	N.d.	^93^
				MTZ (control)_IC50_ = 1.0 μg/mL				
*Thymus zygis* L.	Aerial parts	EO	*T. gallinae*	IC_50_ = 133.1 μg/mL	*In vitro*	Thymol (39.5%)	N.d.	^93^
				MTZ (control)_IC50_ = 1.0 μg/mL				
**Lauraceae**								
*Nectandra megapotamica* (Spreng.) Mez	Leaves	EO	*T. vaginalis* (ATCC 30236)	IC_50_ = 98.7 μg/mL, MIC = 500 μg/mL	*In vitro*	(+)-α-bisabolol (93.7%)	CC_50_ = 71 μg/mL against VERO cells (SI = 0.72); 20–100% of hemolysis in the concentrations tested	^98^
**Lythraceae**								
*Lawsonia inermis* L.	Leaves	HAEX	*T. vaginalis*	Extract from Rudbar-e-Jonub showed 83% growth inhibition	*In vitro*	N.d.	N.d.	^99^
**Myrtaceae**								
*Eugenia gracillima* Kiaersk.	Leaves	EO	*T. vaginalis* (ATCC 30236 and fresh clinical isolates)	MIC: 500 μg/mL, IC_50_: 185.6 μg/mL	*In vitro*	N.d.	CC_50_ = 43.54 μg/mL; (MRC-5 fibroblasts, SI = 0.23), 47.97 μg/mL (HMVII vaginal cells, SI = 0.26); Erythrocytes HC_50_ = 44.67 μg/mL (SI = 0.24)	^100^
*Eugenia pohliana* DC.	Leaves	EO	*T. vaginalis* (ATCC 30236 and fresh clinical isolates)	MIC: 500 μg/mL, IC_50_: 257.8 μg/mL	*In vitro*	δ-cadinene (20.23%)	CC_50_ = 25.45 μg/mL; (MRC-5 fibroblasts, SI = 0.10), 301.9 μg/mL(HMVII vaginal cells, SI = 1.17); Erythrocytes HC_50_ = 296.3 μg/mL (SI = 1.15)	^100^
*Myrciaria floribunda* (H.West ex Willd.) O.Berg.	Leaves	EO	*T. vaginalis* (ATCC 30236 and fresh clinical isolates)	MIC: 500 μg/mL, IC_50_: 162.9 μg/mL	*In vitro*	N.d.	CC_50_ = 112.9 μg/mL; (MRC-5 fibroblasts, SI = 0.69), 56.44 μg/mL (HMVII vaginal cells, SI = 0.35); Erythrocytes HC_50_ = 171.6 μg/mL (SI = 1.05)	^100^
*Psidium guajava* L.	Leaves	EO	*T. gallinae*	LC_50_ = 2.70 μg/mL, LC_90_ = 12.54 μg/mL	*In vitro*	β-caryophyllene (30.2%)	N.d.	^101^
				MTZ (control)_LC50_ = 2.17 μg/mL; _LC90_ = 9.72 μg/mL				
*Psidium myrsinites* DC.	Leaves	EO	*T. vaginalis* (ATCC 30236 and fresh clinical isolates)	MIC: 500 μg/mL, IC_50_: 179.6 μg/mL	*In vitro*	N.d.	CC_50_ = 79.28 μg/mL; (MRC-5 fibroblasts, SI = 0.44), 148.8 μg/mL (HMVII vaginal cells, SI = 0.83); Erythrocytes HC_50_ = 108.0 μg/mL (SI = 0.60)	^100^
**Nitrariaceae**								
*Peganum harmala* L. ★	Seeds	AKEX	*T. gallinae*	MIC = 15 μg/mL (24 h); AKEX at 25 mg/kg reduced parasite burden in infected pigeons after 1 d and led to a full recovery after 3 d	*In vitro/In vivo*	Harmine and harmaline	No mortality or side effects were observed in any of the treated groups	^69^
				MTZ (control)_MIC_ = 50.0 μg/mL				
**Papaveraceae**								
*Argemone mexicana* L.	Leaves, stem	MEX and fractions	*T. vaginalis* (GT15)	MIC = 1,000 μg/mL; IC_50_ = 67.2–70.8 μg/mL; MEX fractions at 125 μg/mL showed lethality rates of 73.0–79.2%	*In vitro*	Quinones, sterols, alkaloids, flavonoids, saponins, triterpenes, and carbohydrates; Berberine and jatrorrhizine	Low hemolysis rates (2.3–2.8%)	^102^
				MTZ (control)_IC50_ = 0.1 μg/mL				
*Papaver rhoeas* L.	Flowers, leaves, capsules	EO, MEX, HEX, EAEX, EtEX, IFEX	*T. gallinae*	EO (flowers) and IFEX (flowers) reduced parasite viability in >90% at 0.4 mg/mL; HEX (capsules) caused 83.2% of mortality with 0.4 mg/mL	*In vitro*	EO (flowers): alkanes (36.3%) and palmitic acid (31%); HEX (capsules): fatty acid esters (42.5%)	N.d.	^82^
**Piperaceae**								
*Piper crocatum* Ruiz and Pav.	Leaves	EtEX	*T. vaginalis*	MIC = 5%	*In vitro*	Alkaloids, flavonoids, polyphenols, quinones, and saponins	N.d.	^103^
*Piper nigrum* L.	Fruit	HEX, EO	*T. vaginalis*	HEX and EO _MIC_ = 156 μg/mL	*In vitro*	EO: β-caryophyllene (40.62%)	EO _CC50_ = 309 μg/mL (24 h) and 257.42 μg/mL (48 h) using VERO cells; SI = 13.2	^104^
**Poaceae**								
*Cymbopogon citratus* (DC.) Stapf	Leaves, stem	EO	*T. vaginalis*	MLC = 0.04% (24 h) and 0.03% (48 h)	*In vitro*	Geranial (43.3%) and neral (30.3%)	CC_50_ = 0.12% in HeLa cells (SI = 3.0, 24 h)	^94^
*Cymbopogon flexuosus* (Nees ex Steud.) W.Watson	Not specified	EO	*T. gallinae*	MLC = 550 μg/mL, MIC = 528 μg/mL, IC_50_ = 220 μg/mL,	*In vitro/In vivo*	β-geranial (45.74%) and Z-citral (34.42%)	No deaths or adverse effects observed in treated Quails *(C. coturnix* japonica)	^105^
				MTZ (control)_IC50_ = 0.1 μg/mL; EO (0.55 mg/kg) eliminated trophozoites 4 days p.i.				
*Cymbopogon nardus* (L.) Rendle ★	Aerial parts	EO	*T. foetus* (B1)	EC_50_ = 0.391 μg/mL (24 h)	*In vitro*	Citronellal (40.1%) and geraniol (25.3%)	N.d.	^70^
				MTZ (control)_EC50_ = 0.016 μg/mL				
**Polypodiaceae**								
*Pleopeltis crassinaervata (*Fée) T. Moore	Fronds, roots, rhizomes	MEX, HEX, fractions	*T. vaginalis* (ATCC 30236)	Subfractions 5 and 6 _IC50_ = 90.9 and 105.4 μg/mL, respectively; MTZ (control)_IC50_ < 4 μg/mL	*In vitro*	Subfraction 6: pregn-16-en-20-one, 3-hydroxy-, [3β,5β]- (44.93%)	No cytotoxicity in Vero (CCL-81) cells up to 200 μg/mL; SI = 4.37 and 3.91 for subfractions 5 and 6, respectively	^62^
**Ranunculaceae**								
*Nigella sativa* L.	Seeds	AEX, EtEX, SO	*T. vaginalis*	AEX and EtEX _MLC_ = 10 mg/mL,	*In vitro*	EtEX: linoleic (41.1%) and oleic acid (21.6%); SO: linoleic (49.4%) and oleic acid (25.7%)	N.d.	^106^
				SO _MLC_ = 2 mg/mL (24 h)				
				MTZ (control)_MLC_ = 50 μg/mL				
**Rhamnaceae**								
*Ziziphus vulgaris* L.	Fruits	EtEX	*T. gallinae*	MIC = 50 μg/mL (24 h)	*In vitro*	Catechin (22.67%)	N.d.	^107^
				MTZ (control)_MIC_ = 50.0 μg/mL				
**Rosaceae**								
*Rosa damascena* Mill.	Petals	HAEX, AEX, RO	*T. vaginalis*	HAEX _IC50_ = 1.41 (24 h) and 1.44 mg/mL (72 h), AEX _IC50_ = 15.24 (24 h) and 5.56 mg/mL (72 h), RO _IC50_ = 1.79 (24 h) and 6.11 mg/mL (72 h)	*In vitro*	Tannins, anthraquinones, and flavonoids were detected in both HAEX and AEX, whereas tannins and flavonoids were present in RO	N.d.	^108^
**Rutaceae**								
*Atalantia sessiflora* Guillaumin	Leaves	EO	*T. vaginalis* (G3)	IC_50_ = 0.016%, IC_90_ = 0.03%, MLC = 0.06%	*In vitro*	Linalool (16.21%)	No cytotoxicity was observed (CC_50_ > 100 μg/mL) in RAW 264.7 macrophages	^109^
*Citrus grandis* (L.) Osbeck	Peel	EO	*T. vaginalis*	MLC = 0.095% (24 h) and 0.06% (48 h)	*In vitro*	Limonene (82%)	CC_50_ = 0.25% using HeLa cells (SI = 2.6, 24 h)	^94^
*Haplophyllum myrtifolium* Boiss	Aerial and underground parts	EtEX, AKEX	*T. vaginalis*	EtEX _MIC and MLC_ = 200 and 400 μg/mL, AKEX _MIC and MLC_ = 400 and 800 μg/mL	*In vitro*	N.d.	N.d.	^110^
				MTZ (control)_MLC_ = 50.0 μg/mL				
**Sapotaceae**								
*Manilkara rufula* (Miq.) H.J.Lam	Leaves	Acetone fractions (M30 and M100)	*T. foetus* (TFK), *T. vaginalis* (ATCC 30236)	M30 and M100 _MIC_ = 1.0 mg/mL (*T. foetus*); MTZ (control)_MIC_ = 0.012 mg/mL	*In vitro*	Prodelphidin, procyanidin dimer, gallic acid, gallocatechin, epigallocatechin, epicatechin, gallocatechin 3-*O*-gallate, epicatechin 3-Ogallate, quercetin 3-*O*-a-rhamnopyranoside, rutin, and other flavonoids	M30 and M100 presented cytotoxicity (>70%) against HMVII and HeLa cells (24 h)	^111^
				M100 at 0.125 mg/mL reduced *T. vaginalis* viability in almost 80%				
	Leaves	H100 saponin fraction	*T. vaginalis* (ATCC 30236 and fresh clinical isolates)	MIC = 0.5 and 1.0 mg/mL against ATCC 30236 and TV-LACM2R, respectively	*In vitro*	Bidesmosidic saponins (Mi-saponin C)	No cytotoxicity against HMVII and HeLa cells (24 h); 100% hemolysis (24 h)	^112^
				MTZ (control)_MIC_ = 0.012 mg/mL (24 h)				
**Scrophulariaceae**								
*Verbascum thapsus* L.	Whole plant	EtEX	*T. vaginalis*	IC_50_ = 73.8 μg/mL (when combined with *Zingiber officinale*); MTZ (control)_IC50_ = 0.03 μg/mL (24 h)	*In vitro*	N.d.	No toxicity against mouse macrophages up to 800 μg/mL (48 h)	^113^
**Solanaceae**								
*Solanum lycopersicum* L.	Leaves, stems, peels	PW	*T. vaginalis* (G3), *T. foetus* (D1)	PW (leaf) reduced 70.0% of *T. vaginalis* and 99.5% of *T. foetus* viability*;* PW (leaf)IC_50_ = 0.02% for *T. vaginalis* and 0.01% for *T. foetus*	*In vitro*	α-tomatine	N.d.	^114^
				MTZ (control)_IC50_ = 0.72 μM (*T. vaginalis*) and 0.49 μM (*T. foetus*)				
*Solanum lycopersicum* var. cerasiforme	Peel	PW	*T. vaginalis* (G3), *T. foetus* (C1 and D1)	PW at 0.02% (w/v) reduced viability in 52.2% (G3), 66.8% (C1), and 81.3% (D1)	*In vitro*	N.d.	N.d.	^115^
**Theaceae**								
*Camellia sinensis* (L.) Kuntze	Leaves	EtEX	*T. gallinae*	MIC = 25 μg/mL (24 h)	*In vitro*	Caffeine (24.07%)	N.d.	^107^
				MTZ (control)_MIC_ = 50.0 μg/mL				
**Zingiberaceae**								
*Amomum tsao-ko Crevost* et Lemaire	Fruits	EO	*T. vaginalis* (Tv1 and Tv2)	MLC and IC_50_ = 44.97 and 22.49 μg/mL (Tv1), 89.93 and 44.97 μg/mL (Tv2)	*In vitro*	Geraniol (13.69%)	N.d.	^116^
*Curcuma longa* L.	Rhizome	EtEX	*T. vaginalis* (GT15)	IC_50_ = 496.36 μg/mL	*In vitro*	Tannins, phenols, and flavonoids	Low hemolysis levels (<5%)	^81^
				MTZ (control)_IC50_ = 0.07 μg/mL				
*Hornstedtia bella* Škorničk	Whole plant, leaves, rhizomes	EO	*T. vaginalis* (G3)	Rhizome IC_50_ = 0.25 (1 h) and 0.004% (48 h), Leaf IC_50_ = 1.0 (1 h) and 0.008% (48 h)	*in vitro*	EO (whole plant): β-pinene (25.52%), EO (leaf): germacrene D (17.47%), EO (rhizome): β-pinene (16.07%)	No cytotoxicity in VERO and MRC-5 cells using rhizome EO; Leaf EO showed weak cytotoxicity (CC_50_ > 80 μg/mL)	^117^
*Zingiber officinale* Roscoe	Whole plant	EtEX	*T. vaginalis*	IC_50_ = 93.8 μg/mL (24 h)	*In vitro*	N.d.	N.d.	^118^
				MTZ (control)_IC50_ = 0.0326 μg/mL				
	Whole plant	EtEX	*T. vaginalis*	IC_50_ = 73.8 μg/mL (when combined with *Verbascum thapsus*); MTZ (control)_IC50_ = 0.03 μg/mL after 24 h	*In vitro*	N.d.	No toxicity against mouse macrophages up to 800 μg/mL (48 h)	^113^
	Rhizomes	EtEX	*T. gallinae*	MIC = 25 μg/mL (24 h)	*In vitro*	α-Curumene (27.51%) and α-gigeberene (22.56%)	N.d.	^92^
				MTZ (control)_MIC_ = 50.0 μg/mL (24 h)				

a AEX: aqueous extract; AKEX: alkaloid extract; CC_50_: 50% cytotoxic concentration; CHEX: chloroform extract; DEX: dichloromethane extract; EAEX: ethyl acetate extract; EC_50_: half-maximal effective concentration; EO: essential oil; EtEX: ethanolic/alcoholic extract; HAEX: hydroalcoholic extract; HC_50_: 50% hemolytic concentration; HEX: hexanic extract; IC_50_: half-maximal inhibitory concentration; IFEX: infusion; LC_50_: median lethal concentration; LC_90_: 90% lethal concentration; MEX: methanolic extract; MIC: minimum inhibitory concentration; MLC: minimum lethal concentration; MTZ: metronidazole; N.d.: not determined; N.r.: not reported; p.i.: post infection; PW: powder; RO: rose oil; SO: seed oil/fixed oil; SI: selectivity index. ★ indicates selected candidates based on potency combined with at least one additional translational criterion, such as selectivity, activity against resistant isolates, mechanistic evidence, or *in vivo* efficacy.

Across the plant-derived products evaluated for antitrichomonal activity, *T. vaginalis* was the most frequently studied species, with 43 products reporting quantitative concentration-based end points. Of these, 20 (46.5%) showed reported activity values ≤ 100 μg/mL, whereas 23 (53.5%) showed values >100 μg/mL. For *T. gallinae*, 21 products reported quantitative values, of which 9 (42.9%) were ≤ 100 μg/mL and 12 (57.1%) were >100 μg/mL. In contrast, quantitative concentration data for *T. foetus* were scarce, with only two products identified, one in each concentration category (50.0%). These proportions are descriptive and do not represent a uniform potency classification, since the original studies employed different activity end points, including IC_50_, EC_50_, MIC, and MLC. Based exclusively on the lowest reported activity concentrations, the plant-derived products that stood out for each trichomonad species were *Hornstedtia bella* and *Elaeodendron trichotomum* against *T. vaginalis* (67), *Artemisia sieberi* (68) and *Peganum harmala* (69) against *T. gallinae*, and *Cymbopogon nardus* (70) and *Caralluma procumbens* (71) against *T. foetus*. Plant species, botanical families, phytochemical composition, trichomonad species evaluated, and additional experimental information are summarized in Table 1 and discussed in the following sections.

#### 2.1.1 Extracts and Fractions

The *Elaeodendron trichotomum* (Celastraceae) extracts emerged among the most active plant-derived preparations identified against *T. vaginalis* in this review. Among six crude extracts prepared from leaves, stem bark, and root bark with dichloromethane and methanol and screened *in vitro* against several protozoan parasites, the dichloromethane extract from the root bark showed the strongest activity against *T. vaginalis* (GT3 isolate), with an IC_50_ of 0.46 μg/mL, compared with 0.12 μg/mL for MTZ under the same experimental conditions. HPLC analysis of this bioactive extract detected the quinonemethide triterpenes tingenone (3.84%) and pristimerin (0.14%). However, because these compounds were present at relatively low levels, the authors suggested that they may not fully account for the pronounced anti-*T. vaginalis* effect, pointing instead to the possible contribution of additional active constituents or synergistic interactions within the extract.^67^

A comparative *in vitro* assay of methanolic extracts from *Quercus infectoria* (Fagaceae), *Pistacia khinjuk* (Anacardiaceae), and *Satureja khuzestanica* (Lamiaceae) showed that all three methanolic extracts inhibited *T. vaginalis* in a concentration- and time-dependent manner. *Q. infectoria* showed the strongest activity (IC_50_ = 3.4 μg/mL at 48 h), followed by *S. khuzestanica* and *P. khinjuk*. For comparison, MTZ showed IC_50_ values of 0.5 and 0.09 μg/mL after 24 and 48 h, respectively, indicating that the plant extracts remained less active than the reference drug despite their pronounced time-dependent increase in activity. Flow cytometry further showed apoptosis-like cell death, particularly after treatment with *Q. infectoria.* ^72^

Aerial-part extracts of *Scutellaria havanensis* (Lamiaceae) also showed relevant *in vitro* activity against *T. vaginalis* (T-H7 isolate). Both the methanolic and chloroform extracts were active, with IC_50_ values of 20.4 and 12.9 μg/mL, respectively, indicating greater potency for the less polar extract. This difference, however, was accompanied by lower selectivity, since cytotoxicity assays in mouse peritoneal macrophages yielded CC_50_ values of 149.6 μg/mL for the methanolic extract and 35.8 μg/mL for the chloroform extract, corresponding to an SI of 7.3 and 2.8, respectively. MTZ, used as the reference control, showed an IC_50_ of 0.62 μg/mL in the same study. Gas chromatography–mass spectrometry (GC-MS) analysis of the chloroform extract identified 74 compounds, predominantly hydroxy- and methoxyflavones, which accounted for approximately 77.96% of the total composition. Among these, wogonin (48.27%) was the major constituent of the extract and may therefore be an important contributor to the observed anti-*T. vaginalis* activity.^96^ The antitrichomonal activity of wogonin as an isolated compound is further discussed in the Isolated Compounds section.

Fakhrieh-Kashan et al. 2018 ^113^ studied an alcoholic extract prepared from a combination of *Verbascum thapsus* (Scrophulariaceae) and *Zingiber officinale* (Zingiberaceae) against *T. vaginalis*. Although complete parasite elimination required the highest tested concentration (800 μg/mL) after 48 h, the extract reached an IC_50_ of 73.8 μg/mL at 24 h, whereas MTZ showed an IC_50_ of 0.03 μg/mL under the same conditions, indicating substantially greater potency of the reference drug. Flow cytometry further indicated that its antitrichomonal effect was mainly associated with programmed cell death rather than nonspecific damage, since apoptosis rates increased from 18.97 to 77.19% across concentrations of 25–400 μg/mL after 48 h, whereas necrosis remained low (1.16–4.09%). In parallel, cytotoxicity assays using mouse peritoneal macrophages showed minimal toxicity, suggesting a favorable safety profile for this combined extract.

Extracts from the aerial parts of *Pelargonium quercetorum* (Geraniaceae) likewise displayed antiparasitic activity against *T. vaginalis*. The extract prepared by maceration using ethyl acetate was the most effective, yielding an IC_50_ of 89 μg/mL and an MLC of 250 μg/mL after 48 h of incubation. For comparison, MTZ showed an MLC of 3.1 μg/mL in the same assay. Activity varied according to the extraction solvent, with the ethyl acetate extract showing the strongest effect, followed by the hexane extract, whereas the aqueous preparation was markedly less active. Because the active metabolites were not chemically characterized, the compounds responsible for the observed effect remain undetermined.^89^

A bioguided fractionation study of *Pleopeltis crassinervata* (Polypodiaceae) by Anacleto-Santos et al. 2024 ^62^ further illustrates how activity can be concentrated into less polar fractions. When extracts, fractions, and subfractions obtained from the fronds were tested against *T. vaginalis* (ATCC 30236 isolate), the hexane fraction of the methanolic extract emerged as the most active preparation, with an IC_50_ of 82.3 μg/mL after 24 h. Subsequent chromatographic separation generated seven subfractions, among which Hsf5 and Hsf6 retained the strongest activity, with IC_50_ values of 90.9 μg/mL and 105.4 μg/mL, respectively. In comparison, MTZ showed an IC_50_ below 4 μg/mL, indicating that the active subfractions remained considerably less active than the reference drug. Both subfractions also showed moderate selectivity toward the parasite, as indicated by CC_50_ values of 394.3 μg/mL for Hsf5 and 416.6 μg/mL for Hsf6 in Vero cells, corresponding to an SI of 4.37 and 3.91. GC-MS analysis of Hsf6 revealed a predominance of terpenoid-related constituents, particularly 16-pregnenolone (44.9%), followed by bis­(2-ethylhexyl) phthalate (17.9%) and 1(10),9(11)-B-homolanistadiene (11.7%), together with other terpenoid and aliphatic compounds such as α-cadinol and β-sitosterol acetate. Taken together, these data suggest that terpenoid-rich fractions from *P. crassinervata* are associated with the anti-*T. vaginalis* effect observed against *T. vaginalis*.

A clear solvent-dependent pattern was also observed for *Juniperus communis* (Cupressaceae) fruit extracts tested against several protozoan parasites, including an MTZ-resistant isolate of *T. vaginalis* (ATCC50143). Among the preparations evaluated, only the chloroform extract showed relevant antitrichomonal activity, with an IC_50_ of 30 μg/mL, whereas the ethanol, methanol, and aqueous extracts were substantially less effective, all with IC_50_ values above 250 μg/mL. This marked difference suggests that the compounds responsible for activity are concentrated in the lipophilic fraction, in line with previous reports describing terpenoids and other nonpolar secondary metabolites with antimicrobial properties in *J. communis* fruits.^85^

A broader screening of methanolic extracts from seven Lamiaceae species was performed by Tüzün et al. 2022.^97^ Extracts prepared from the aerial parts of *Phlomis armeniaca*, *Phlomis pungens* var. *pungens*, *Phlomis sieheana*, *Thymus haussknechtii*, *Thymus kotschyanus* var. *kotschyanus*, *Stachys lavandulifolia* subsp. *lavandulifolia*, and *Salvia verticillata* subsp. *verticillata* were evaluated *in vitro* against *T. vaginalis* (ADUTR011 isolate) at concentrations ranging from 250 to 1.9 μg/mL. Among them, only *T. haussknechtii* showed clear trichomonacidal activity, reaching both MIC and MLC values of 62.5 μg/mL after 48 h of incubation, whereas the remaining species were inactive under the tested conditions. MTZ showed a MIC of 15.6 μg/mL under the same experimental conditions, indicating approximately 4-fold greater activity than the plant extract based on this end point. Even so, this study underscored the variability of anti-*T. vaginalis* activity even within the same botanical family.

Methanolic extracts from both stems and leaves of *Argemone mexicana* (Papaveraceae) also showed relevant antitrichomonal activity against *T. vaginalis* (GT15 isolate). The two preparations inhibited trophozoite growth in a dose-dependent manner, with very similar IC_50_ values of 70.8 μg/mL for the stem extract and 67.2 μg/mL for the leaf extract. For comparison, MTZ showed an IC_50_ of 0.1 μg/mL in the same study. Chemical analysis of the most active fractions by chromatographic and mass spectrometric methods indicated the presence of the alkaloids berberine and jatrorrhizine, both previously recognized for their antiparasitic potential. Additionally, the crude extracts showed very low toxicity in human erythrocytes, with hemolysis remaining below 3% even at 1000 μg/mL, which supports a favorable preliminary safety profile for these preparations.^102^ Berberine is further discussed in the section on Isolated Compounds, where its direct anti-*T. vaginalis* activity is evaluated.

Arbabi et al. 2016 ^118^ showed that the ethanolic rhizome extract of *Zingiber officinale* (Zingiberaceae) exhibited moderate *in vitro* activity against *T. vaginalis*, with an IC_50_ of 93.8 μg/mL after 24 h of incubation. Under the same conditions, MTZ showed an IC_50_ of 0.0326 μg/mL, indicating that the extract required substantially higher concentrations to achieve a comparable inhibitory end point. Although complete loss of trophozoite viability was only achieved at the highest tested concentration (800 μg/mL) after 48 h, the extract displayed very low toxicity toward mouse peritoneal macrophages, with mortality rates ranging from 0.11 to 0.31% across concentrations of 25 to 800 μg/mL. Flow cytometry further indicated that its trichomonacidal effect was associated with apoptosis in a clear concentration-dependent manner, with total apoptotic rates increasing from 17% at 25 μg/mL to 100% at 800 μg/mL after 48 h, whereas the untreated control showed only 2.9% apoptosis.

The antiparasitic activity of the methanolic extract obtained from the leaves of *Kalanchoe daigremontiana* (Crassulaceae) and its major flavonoid constituent, quercetin, was evaluated against *T. vaginalis* (GT15 isolate) trophozoites. The methanolic leaf extract inhibited parasite growth with an IC_50_ value of 105.27 μg/mL, while quercetin exhibited markedly higher activity, presenting an IC_50_ of 21.17 μg/mL. MTZ, used as the reference drug, showed an IC_50_ of 0.17 μg/mL under the same experimental conditions. Hemolysis assays indicated low toxicity for the crude extract, with values below 7.2%, while quercetin itself showed negligible hemolytic activity and even exerted a cytoprotective effect on erythrocytes. These findings support the view that flavonoids, particularly quercetin, are likely important contributors to the antiparasitic activity of *K. daigremontiana* methanolic extract.^84^

Fractions obtained from the fruits of *Poincianella microphylla* (Fabaceae) provided another example of activity associated with polyphenol-rich preparations. After fractionation of the aqueous extract, fraction M5 was classified as highly active against *T. vaginalis* (ATCC 30236 isolate), with an IC_50_ of 70.41 μg/mL after 24 h of incubation, whereas fraction M10 showed lower activity, with an IC_50_ of 142.1 μg/mL. LC-DAD-MS/MS profiling detected 26 metabolites and indicated that hydrolyzable tannins were the predominant constituents, including O-digalloyl hexoside, O-(digalloyl) quinic acid, tri-*O*-galloyl-hexahydroxydiphenoyl (HHDP)-hexoside, geraniin isomers, epicatechin, ellagic acid, and procyanidin dimers. Correlation analysis further linked several of these tannins (particularly O-galloyl-HHDP-dehydrohexahydroxydiphenoyl (DHHDP)-hexoside, O-digalloyl-HHDP-hexoside, O-(digalloyl) quinic acid, and O-digalloyl hexoside) to the observed anti-*T. vaginalis* effect. Importantly, the active fractions showed no hemolytic activity in human erythrocytes and caused no significant mortality in the *Galleria mellonella* invertebrate model, supporting the view that hydrolyzable tannins from *P. microphylla* are relevant sources of bioactive compounds for further investigation against human trichomoniasis.^88^

One of the most active preparations identified with anti-*T. gallinae* activity was the alkaloid extract from *Peganum harmala* seeds (Zygophyllaceae). This fraction, obtained from ethanolic seed extracts and characterized by HPLC, contained the β-carboline alkaloids harmine and harmaline and showed pronounced *in vitro* activity, with a MIC of 15 μg/mL after 24 h of incubation, while MTZ showed a reported MLC of 50 μg/mL in the same study. Its efficacy was further supported *in vivo*, since oral treatment of experimentally infected pigeons with 2.5 mg/100 g body weight for three consecutive days led to complete recovery and elimination of trophozoites, whereas MTZ did not achieve full parasite clearance within the same period. No mortality or adverse effects were observed in treated birds, indicating that the extract combined high activity with good tolerability in this avian model.^69^ The individual activities of the major β-carboline alkaloids harmine and harmaline are further discussed in the section on Isolated Compounds.

A related *in vitro* assay with an alcoholic extract from the flowering branches of *Lavandula angustifolia* (Lamiaceae) also showed activity against *T. gallinae*, although at a lower potency than that observed for *Peganum harmala*. GC-MS analysis indicated that the extract was dominated by linalool (26.20%) and borneol (22.70%), two monoterpenoid constituents commonly associated with antimicrobial effects. Under the tested conditions, the extract reached a MIC of 50 μg/mL after 24 h of incubation, identical to the MIC reported for MTZ in the same assay, placing *L. angustifolia* among the moderately active plant-derived products evaluated against the avian parasite.^92^

*Zingiber officinale* was investigated against *T. gallinae*, and its alcoholic rhizome extract showed a pronounced activity.^92^ Tested at concentrations ranging from 5 to 100 μg/mL against trophozoites isolated from infected pigeons, the extract reached a MIC of 25 μg/mL after 24 h, whereas MTZ required 50 μg/mL under the same conditions. Under these experimental conditions, the lower MIC of the extract relative to MTZ indicates a stronger *in vitro* inhibitory effect against *T. gallinae*. GC-MS analysis indicated a profile rich in ginger-derived terpenoids and phenolic constituents, mainly α-curumene (27.51%), α-zingiberene (22.56%), and gingerol (19.67%). Taken together, these results suggest that *Z. officinale* may be more active against *T. gallinae* and represents a potential source of compounds for the control of avian trichomoniasis.^92^

Rahimi et al. 2024 ^107^ demonstrated that the ethanolic extracts from *Camellia sinensis* (Theaceae) leaves and *Ziziphus vulgaris* (Rhamnaceae) fruits were likewise active against *T. gallinae*. GC-MS analysis showed that the *C. sinensis* extract was dominated by caffeine (24.07%), whereas the *Z. vulgaris* extract was characterized mainly by phenolic and flavonoid constituents, especially catechin (22.67%). In *in vitro* assays, *C. sinensis* showed the stronger early effect, with a MIC of 25 μg/mL after 24 h, while *Z. vulgaris* reached a MIC of 50 μg/mL under the same conditions. In comparison, the MTZ control showed a MIC of 50 μg/mL, possibly indicating greater activity for *C. sinensis* extract. Both extracts became more effective with longer exposure, achieving complete inhibition of trophozoites at lower concentrations after 72 h, which indicates a clear time-dependent enhancement of activity.

A comparable pattern was observed for ethanolic extracts prepared from *Q. infectoria* galls (Fagaceae) and *Ceratonia siliqua* pods (Fabaceae), both of which inhibited *T. gallinae* trophozoites in a dose-dependent manner. GC-MS analysis showed that the *Q. infectoria* extract was characterized mainly by β-pinene oxide (21.09%) and β-pinene (14.31%), whereas the *C. siliqua* extract contained predominantly nonadecane (23.34%) and 1,2-benzenedicarboxylic acid dibutyl ester (15.95%). Among the two, *Q. infectoria* displayed the stronger early activity, reaching a MIC of 25 μg/mL after 24 h, whereas *C. siliqua* required 50 μg/mL to fully suppress parasite motility. Under the same conditions, MTZ showed a MIC of 50 μg/mL, indicating that *Q. infectoria* achieved complete inhibition at a lower concentration, while *C. siliqua* showed activity comparable to the reference drug based on this end point.^86^

For *Manilkara rufula* (Sapotaceae), the available evidence points to more than one active chemical class associated with antitrichomonad effects. In the first study, fractions obtained from leaves of this endemic Caatinga species were evaluated against both *T. vaginalis* (ATCC 30236) and *T. foetus* (TFK). Initial screening of crude acetone fractions at 1.0 mg/mL reduced *T. vaginalis* viability by more than 90% and completely abolished *T. foetus* viability. After Sephadex LH20 fractionation of ML-A10, fractions M30 and M100 remained particularly active and fully reduced *T. foetus* viability, for which the MIC was established at 1.0 mg/mL. However, MTZ showed a markedly lower MIC of 0.012 mg/mL under the same conditions. In addition, both fractions displayed substantial cytotoxicity (>70%) toward HMVII and HeLa cells, indicating limited selectivity despite their antitrichomonal activity. Although none of the tested concentrations fully inhibited *T. vaginalis*, chemical analyses by LC-DAD-MS/MS and MALDI-MS/MS revealed that these active fractions were rich in flavonoids and condensed tannins, including proanthocyanidins in fraction M100. These findings suggest that polyphenol-rich fractions from *M. rufula* may be particularly relevant against the cattle pathogen *T. foetus*.^111^

A subsequent study on the same species shifted attention from polyphenols to saponin-enriched fractions and showed that *M. rufula* also contains bioactive trichomonacidal constituents against *T. vaginalis*. An enriched fraction obtained by Sephadex LH20 chromatography (H100) completely inhibited parasite viability after 24 h with an MIC of 0.5 mg/mL. Kinetic assays indicated a rapid reduction in trophozoite viability within the first hours of exposure, culminating in total suppression after 24 h. LC-ESI-MS, MALDI-MS, and NMR analyses identified several bidesmosidic triterpenoid saponins, including the uncommon Mi-saponin C, and purified saponin-containing fractions reproduced the same complete loss of parasite viability at 0.5 mg/mL, confirming these metabolites as the active constituents. Importantly, the active fraction was not toxic to human vaginal epithelial (HMVII) or HeLa cells at the effective concentration and also potentiated the activity of MTZ against an MTZ-resistant *T. vaginalis* isolate, reinforcing the potential of *M. rufula* saponins as selective trichomonacidal agents and possible adjuvants in therapy.^112^

Unfortunately, there is scarce information regarding the anti-*T. foetus* activity of organic plant extracts. The methanolic stem extract of *Caralluma procumbens* (Apocynaceae) showed *in vitro* antiprotozoal activity, despite requiring concentrations in the mg/mL range. Preliminary phytochemical screening revealed a chemically diverse profile including tannins, alkaloids, glycosides, phenols, flavonoids, quinones, terpenoids, coumarins, and saponins. Complete inhibition of parasite viability was achieved after 24 h at 5 and 10 mg/mL, while 2 mg/mL reduced the viability by about 80% after 48 h. Although this potency was clearly lower than that observed for the most active products identified in the review, the extract showed an effect comparable to MTZ in the parallel assays, suggesting that *C. procumbens* contains metabolites able to significantly impair *T. foetus* viability *in vitro*.^71^

#### 2.1.2 Essential Oils

Donadu et al. 2020 ^117^ evaluated the activity of *Hornstedtia bella* (Zingiberaceae) essential oil (EO) against *T. vaginalis* (G3 isolate). The EO obtained by hydrodistillation from leaves, rhizomes, and the whole plant was analyzed by GC-MS and consisted mainly of monoterpene and sesquiterpene hydrocarbons, with β-pinene, E-β-caryophyllene, α-humulene, germacrene D, and viridiflorene among the principal constituents, although their relative proportions varied according to the plant part. All three oils were active against the G3 isolate, but the rhizome oil was clearly the most active, reaching IC_50_, IC_90_, and MLC values of 0.004, 0.008, and 0.016% (v/v), respectively, after 48 h of incubation. This high activity was accompanied by a favorable preliminary safety profile, since only the leaf oil showed weak cytotoxicity toward mammalian cells, whereas the rhizome and whole plant EO were not cytotoxic to Vero and MRC-5 cells.

Another Zingiberaceae EO, obtained from the fruits of *Amomum tsao-ko*, also displayed *in vitro* activity against *T. vaginalis*. When tested against two clinical isolates (Tv1 and Tv2), the EO produced IC_50_ values of 22.49 and 44.97 μg/mL, respectively, with corresponding MLC of 44.97 and 89.93 μg/mL. Transmission electron microscopy analysis showed marked ultrastructural alterations in treated trophozoites, including damage consistent with disruption of cellular membranes and intracellular organelles.^116^

A relevant anti-*T. vaginalis* profile was reported for the fruit EO of *Trachyspermum ammi* (Apiaceae). GC–MS analysis indicated a composition strongly dominated by thymol (47.05%), followed by γ-terpinene (26.78%) and p-cymene (22.06%). After 48 h of incubation, the oil reached an IC_50_ of 69.47 μg/mL, placing it among the more active EOs identified in this review and reinforcing the recurrent association of thymol-rich monoterpene profiles with antitrichomonal activity.^76^

A distinct chemical pattern was observed in the EOs of *Nectandra* species (Lauraceae), particularly in the leaf oil of *Nectandra megapotamica*. GC-MS analysis across the investigated species identified 73 volatile constituents, mainly monoterpenes, sesquiterpenes, and phenylpropanoid derivatives, but the sample designated ML1 stood out for being dominated by the oxygenated sesquiterpene (+)-α-bisabolol, which accounted for 93.7% of the EO. This EO showed notable activity against *T. vaginalis* (ATCC 30236 isolate), with an IC_50_ of 98.7 μg/mL; parasite viability was already markedly reduced at 250 μg/mL, and complete growth inhibition was achieved at 500 μg/mL. However, its biological profile was less favorable in terms of safety, since the EO also showed cytotoxicity toward Vero cells (CC_50_ = 71 μg/mL), corresponding to a selectivity index of only 0.72, together with pronounced hemolytic activity in human erythrocytes. These findings indicate limited selectivity for the parasite over mammalian cells.^98^

A comparable level of activity was described by Sena-Lopez et al. 2018 ^87^ for the EO of Brazilian red propolis, obtained from the resinous exudates of *Dalbergia ecastophyllum*. GC-MS analysis identified methyl eugenol (13.1%) as the major volatile constituent. Against *T. vaginalis* (ATCC 30236 isolate), the EO reached an IC_50_ of 100 μg/mL, and complete trophozoite death was achieved at 500 μg/mL after 24 h of exposure. Growth-kinetic analysis showed that this concentration reduced parasite viability by about 36% after 12 h and led to total mortality by 24 h, indicating a progressive but effective trichomonacidal action. Cytotoxicity assays in CHO-K1 cells yielded a CC_50_ of 266 μg/mL, suggesting moderate selectivity for the parasite.^87^

The EO of *Dracocephalum kotschyi* (Lamiaceae) was obtained by hydrodistillation and characterized by GC-MS, containing 11 constituents accounting for about 91.5% of the total composition, with copaene (22.15%), methyl geranate (16.31%), and geranial (13.78%) as the major compounds. *In vitro*, the oil inhibited *T. vaginalis* trophozoites in a concentration-dependent manner, reaching an IC_50_ of 84.07 μg/mL after 24 h of exposure. Cytotoxicity assays in J774.A1 hematopoietic cells yielded a CC_50_ of 118.6 μg/mL after 24 h, corresponding to a calculated selectivity index of 1.41, indicating only modest selectivity under these conditions. Flow cytometry with Annexin V–FITC further showed that the EO induced apoptosis-like cell death in the parasite, with apoptotic rates of 9.42 and 13.59% at 100 and 700 μg/mL after 48 h, respectively, indicating that its antitrichomonal effect is at least partly associated with programmed cell death rather than nonspecific toxicity.^91^

The Brazilian Caatinga biome represents a vast source of new biologically active molecules against parasitic diseases. Among seven EOs obtained from Myrtaceae species native to the Brazilian Caatinga, four showed marked activity against *T. vaginalis*: *Eugenia gracillima*, *Eugenia pohliana*, *Myrciaria floribunda*, and *Psidium myrsinites*, all of which completely inhibited trophozoite viability at 500 μg/mL. Although the EOs generally showed moderate toxicity toward mammalian cells, *E. pohliana* showed the most favorable selectivity profile in vaginal epithelial cells and erythrocytes, with selectivity indices of 1.17 and 1.15, respectively. However, its selectivity was markedly lower in MRC-5 fibroblasts (SI = 0.10), indicating that the apparent safety profile was strongly dependent on the host-cell model evaluated. GC-MS analysis of this EO identified d-cadinene (20.23%) and bicyclogermacrene (13.73%) as the major constituents.^100^

A more recent study by Margarita et al. 2025 ^94^ expanded this line of investigation by testing EO from *Cymbopogon citratus* (Poaceae), *Mentha arvensis* (Lamiaceae), and *Citrus grandis* (Rutaceae) against 30 clinical isolates of *T. vaginalis* obtained from symptomatic women in Italy and Vietnam. Among the three oils, *C. citratus* was the most active, with a median MLC of 0.04% after 24 h and 0.03% after 48 h. GC-MS analysis showed that this EO contained 30 constituents, largely dominated by neral and geranial, which together accounted for more than 73% of the total composition. Particularly relevant from a translational perspective, cytotoxicity assays in HeLa cells yielded selectivity indices of 3.0, 4.5, and 2.6 for *C. citratus*, *M. arvensis*, and *C. grandis*, respectively. The EOs did not inhibit the growth of beneficial vaginal bacteria *Lactobacillus crispatus* and *Lactobacillus gasseri*. In addition, susceptibility to the EO was not affected by the presence of the endosymbionts *Mycoplasma hominis* and *Candidatus* Mycoplasma girerdii in the parasite isolates, suggesting that the observed activity is robust across distinct clinical backgrounds.^94^

Regarding avian trichomoniasis, one of the most active EOs was obtained from the aerial parts of *Artemisia sieberi* (Asteraceae). Gas chromatographic analysis showed a monoterpenoid-rich composition dominated by α-thujone (31.5%). This oil displayed strong *in vitro* activity against *T. gallinae*, with a MIC of 10 μg/mL after 24 h, compared with 20 μg/mL for MTZ under the same experimental conditions. Additionally, its efficacy was further confirmed *in vivo*. In experimentally infected pigeons, treatment at 50 mg/kg led to complete recovery after 4 days, whereas MTZ at the same dose required 5 days to eliminate trophozoites. No mortality or clinical adverse effects were observed, indicating that *A. sieberi* EO combined high trichomonacidal activity with good tolerability under the tested conditions.^68^

Akbarian et al. 2024 ^101^ identified a similarly strong *in vitro* effect for the leaf EO of *Psidium guajava* (Myrtaceae) against *T. gallinae*. The oil was characterized by GC-MS as a complex mixture of 31 compounds accounting for 99.5% of the total composition, with β-caryophyllene (30.2%) and α-pinene (18.3%) as the dominant constituents. Its activity was both concentration- and time-dependent, and concentrations of 50, 25, and 12.5 μg/mL caused more than 90% reduction in trophozoite viability after only 12 h of exposure. Probit analysis yielded LC_50_ and LC_90_ values of 2.70 and 12.54 μg/mL, respectively, which were close to those obtained for MTZ (2.17 and 9.72 μg/mL, respectively), indicating a broadly comparable level of activity under the experimental conditions.^101^ The activities of its major constituents, β-caryophyllene and α-pinene, are further discussed in the section on Isolated Compounds.

The EO of *Pelargonium roseum* (Geraniaceae), obtained from the aerial parts by hydrodistillation, also showed relevant activity against *T. gallinae* in both *in vitro* and *in vivo* assays. GC-MS analysis identified β-citronellol (29%) and geraniol (18.5%) as the major constituents of the oil. In *in vitro* assays, the EO reached a 24 h MIC of 20 μg/mL, whereas MTZ showed a MIC of 10 μg/mL under the same conditions. Its efficacy was further supported in experimentally infected pigeons, in which treatment at 50 mg/kg resulted in complete recovery after 5 days, with no mortality or detectable clinical adverse effects. Although slightly less active than MTZ *in vitro*, *P. roseum* EO still demonstrated a consistent antiparasitic effect and good tolerability in the avian model of infection.^90^

An *in vitro* screening of EOs from Lamiaceae and Asteraceae species further reinforced the relevance of terpenoid-rich volatile fractions against *T. gallinae.* ^93^ In that study, 36 EOs obtained by hydrodistillation or steam distillation from 17 species belonging to the genera *Lavandula*, *Salvia*, *Thymus*, *Origanum*, *Satureja*, *Mentha*, *Rosmarinus*, *Ditrichia*, and *Santolina* were evaluated against trophozoites. The most active EOs were those from *Lavandula luisieri*, *Salvia officinalis*, *S. hibrida*, *S. sclarea*, *Thymus vulgaris*, *T. zygis*, *Origanum majorana*, and *Satureja montana*, all with IC_50_ values generally below 200 μg/mL. GC-MS analysis of these more active EO consistently showed predominance of monoterpenes and sesquiterpenes, particularly thymol, p-cymene, γ-terpinene, linalyl acetate, and camphene, suggesting that these compounds are recurrently associated with the anti-*T. gallinae* effect observed across different botanical sources.^93^ Nevertheless, the IC_50_ values of these EOs remained substantially higher than that of MTZ (1.0 μg/mL), indicating considerably lower potency than the reference drug despite their measurable anti-*T. gallinae* activity.

Finally, evidence for *T. foetus* also included a highly active EO from *Cymbopogon nardus* (Poaceae), commonly known as citronella grass. The EO obtained by hydrodistillation from the aerial parts was chemically characterized by gas chromatography and found to be rich in citronellal (40.1%), geraniol (25.3%), and citronellol (12.7%). Against the B1 isolate, it produced a clear dose-dependent effect, with complete trophozoite killing at higher concentrations after 24 h and an EC_50_ of approximately 0.391 μg/mL. Although MTZ remained more active under the same conditions (EC_50_ = 0.016 μg/mL), the citronella oil still displayed high *in vitro* activity, highlighting its terpenoid-rich composition as a relevant source of bioactive compounds for further investigation against bovine trichomoniasis.^70^

Despite their notable antitrichomonal activity, EOs present additional challenges for therapeutic development because their chemical composition may vary according to the botanical source, plant part, environmental conditions, and extraction procedures.^101,117^ Such variability reinforces the need for rigorous chemical standardization and stability assessment before biological results can be considered to be reproducible. Furthermore, the available selectivity data show that high antitrichomonal activity does not necessarily correspond to an adequate safety margin, reinforcing the need to consider host-cell toxicity and selectivity together with antiparasitic activity.

#### 2.1.3 Other Products

In addition to extracts and EOs, powdered plant materials have also been investigated as sources of antitrichomonal compounds. Tomato-derived products are particularly noteworthy in this regard. Building on earlier evidence that tomato glycoalkaloids are active against *T. vaginalis* and bovine *T. foetus*,^119^ Tam et al. 2021 ^114^ evaluated powders prepared from leaves, stems, and peels collected at different ripening stages. After drying and grinding, these materials showed variable effects against both parasites, with leaf powder standing out as the most active preparation. It reduced *T. vaginalis* viability by 70.0 ± 11% and bovine *T. foetus* by 99.5 ± 2.4%, corresponding to IC_50_ values of 0.02% (w/v) and 0.01% (w/v), respectively. The authors associated this activity with the high α-tomatine content detected by HPLC, since leaf and green peel powders contained the highest levels of this glycoalkaloid and, accordingly, green tomato peel powder also showed strong reduction of *T. foetus* viability.

This line of investigation was later extended by the same group to cherry tomato peel powders obtained from seven commercially available varieties grown under organic or nonorganic conditions.^115^ Although most preparations retained antitrichomonal activity against both *T. vaginalis* and *T. foetus*, the magnitude of the effect varied considerably among tomato varieties. Six of the seven powders were active, but one variety lacked activity against *T. vaginalis*, and another was ineffective against *T. foetus*. Among the active samples, powder from variety D produced the strongest reduction of *T. foetus* (81.3%) viability, whereas variety C was the most effective against *T. vaginalis* (66.7%). The authors noted that these tomato varieties were not derived from the same cultivar and that genetic differences likely influenced their glycoalkaloid composition and, consequently, their antiparasitic activity.^115^ Nevertheless, the absence of host-cell toxicity, selectivity, and *in vivo* efficacy data for these powdered preparations currently limits assessment of their therapeutic relevance despite their measurable *in vitro* activity.

### 2.2 Isolated Compounds from Plants with Trichomonacidal Activity

In addition to crude extracts and fractions, isolated natural compounds have also been investigated as potential antitrichomonal agents. These compounds are summarized in Table 2 and encompass alkaloids, flavonoids, terpenes, phenolics, glycoalkaloids, quinines, and sterols.

**Table 2:**
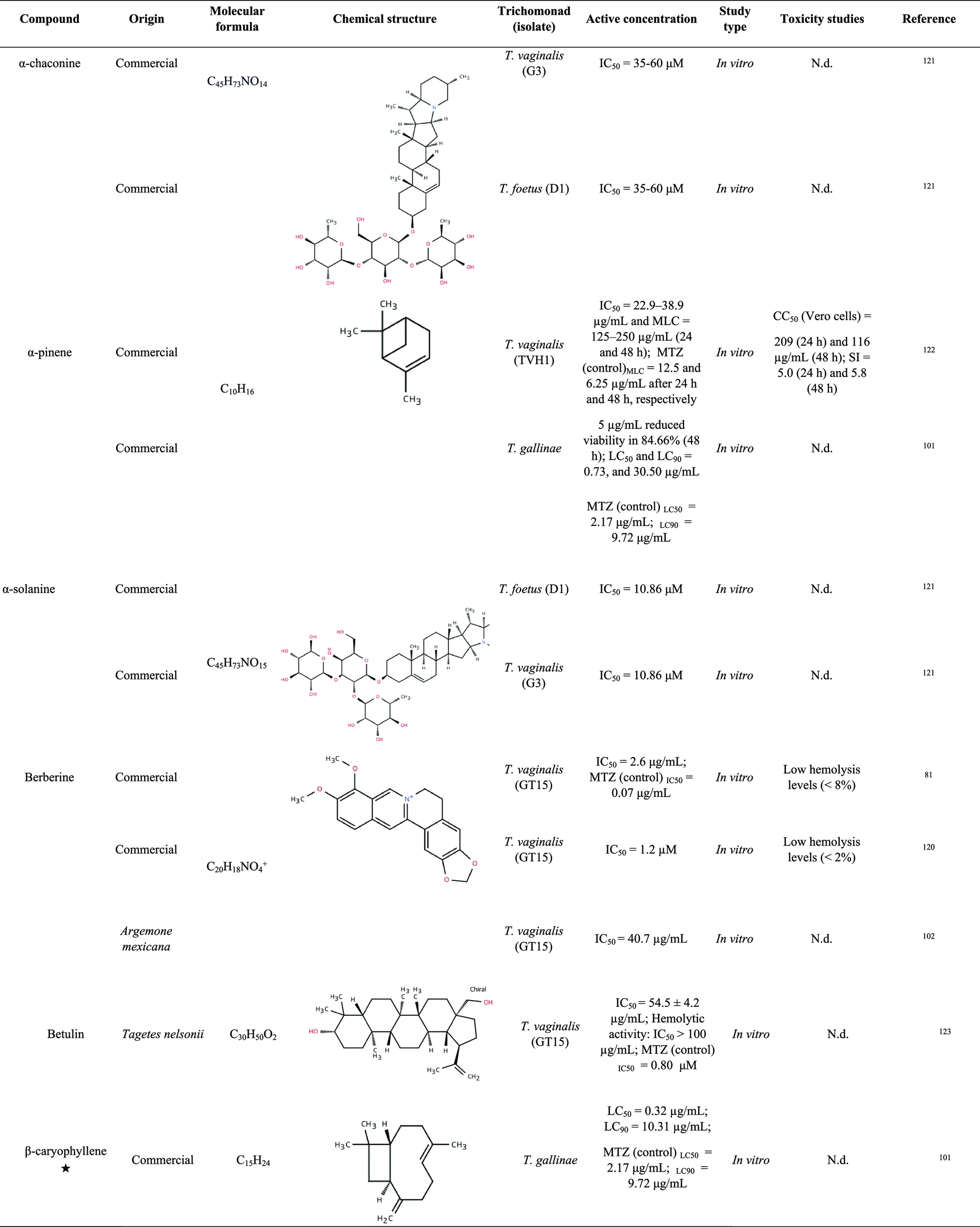

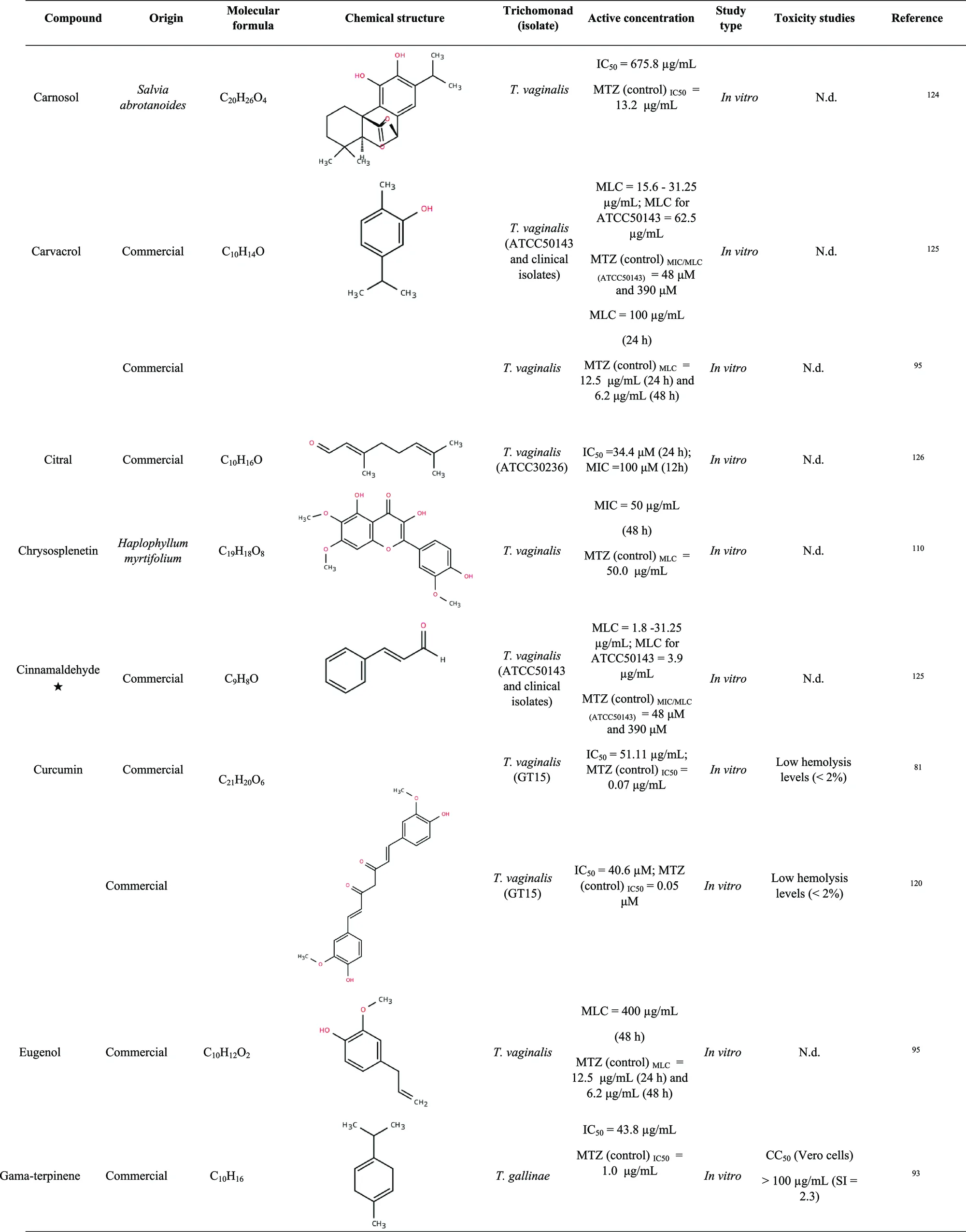

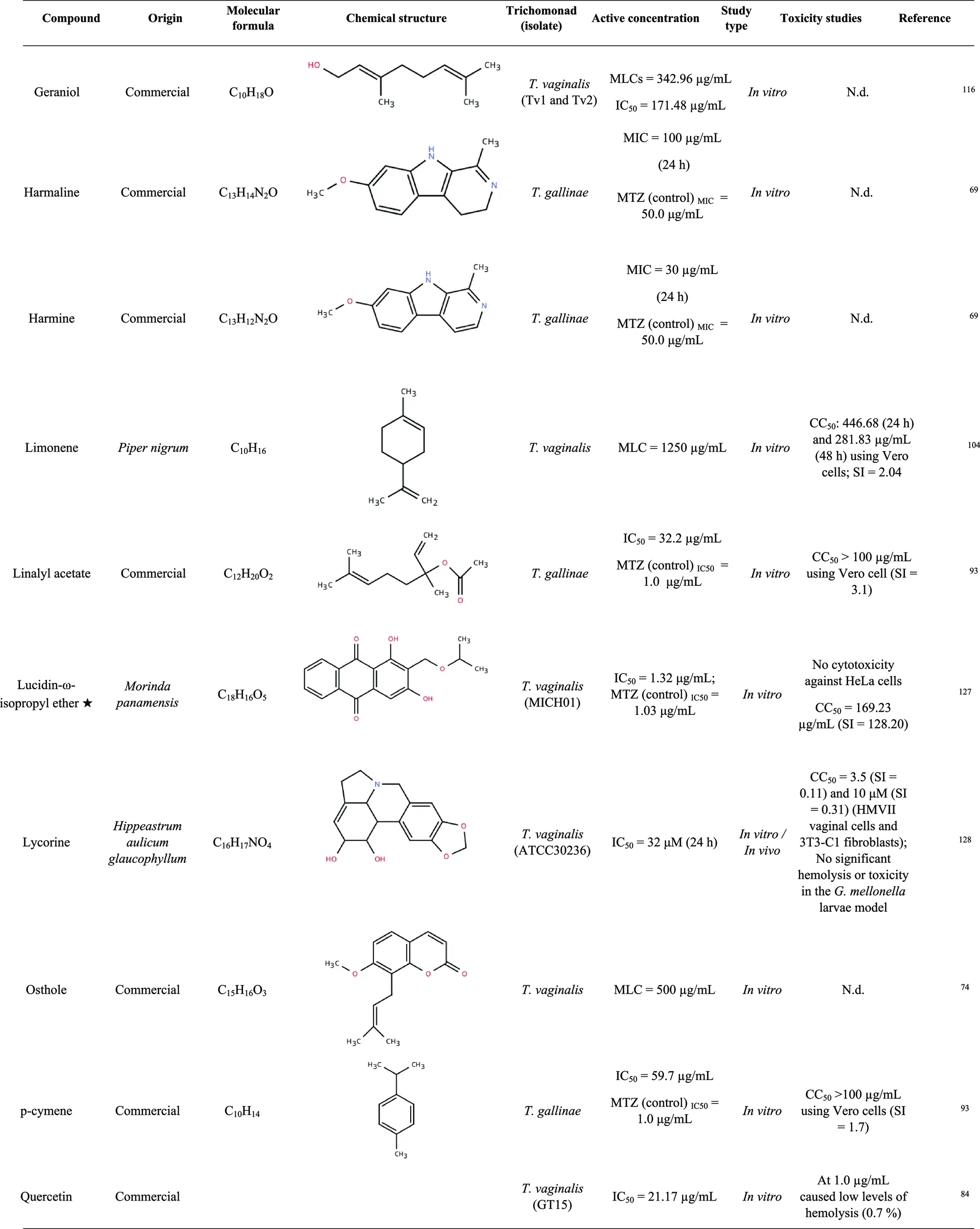

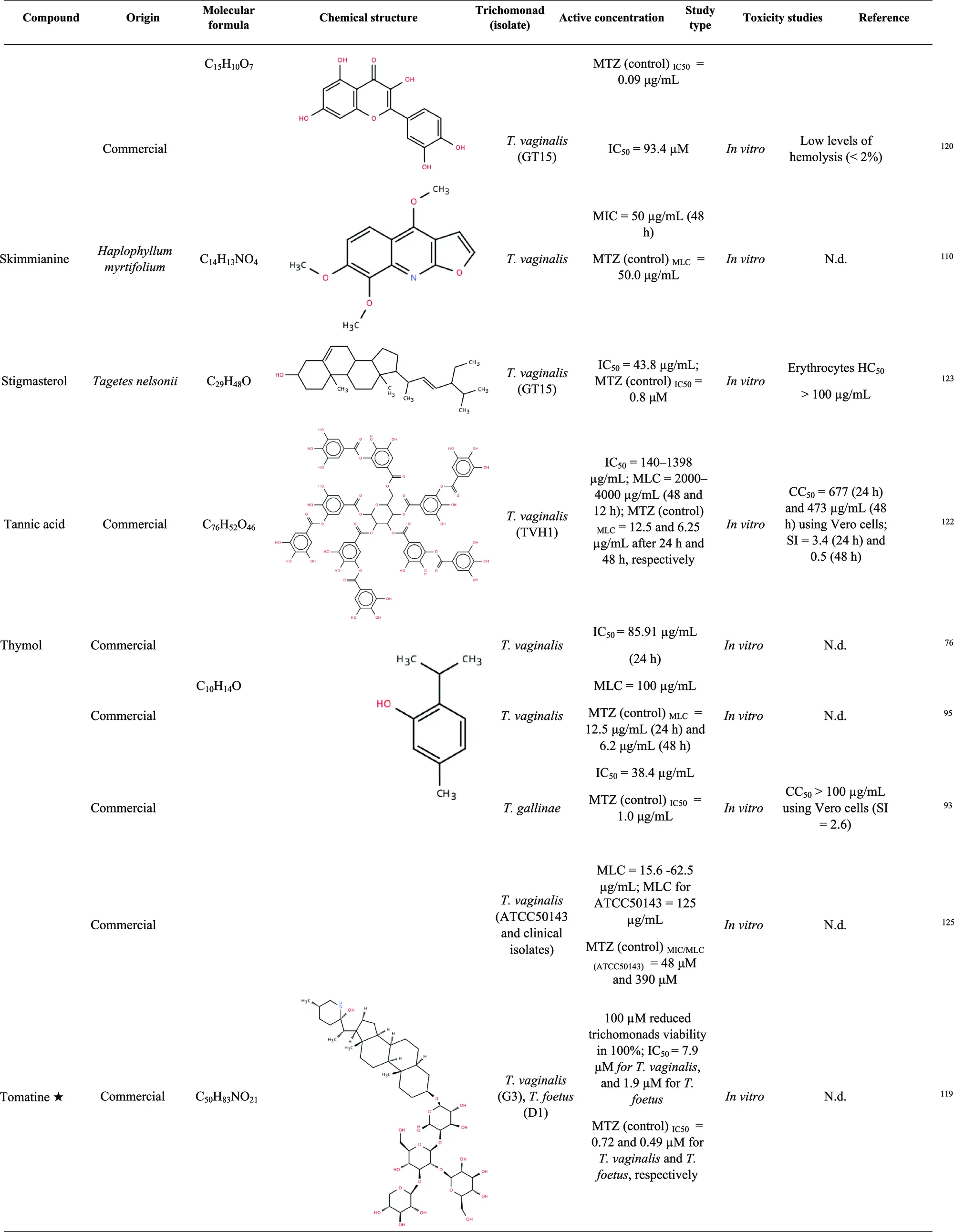

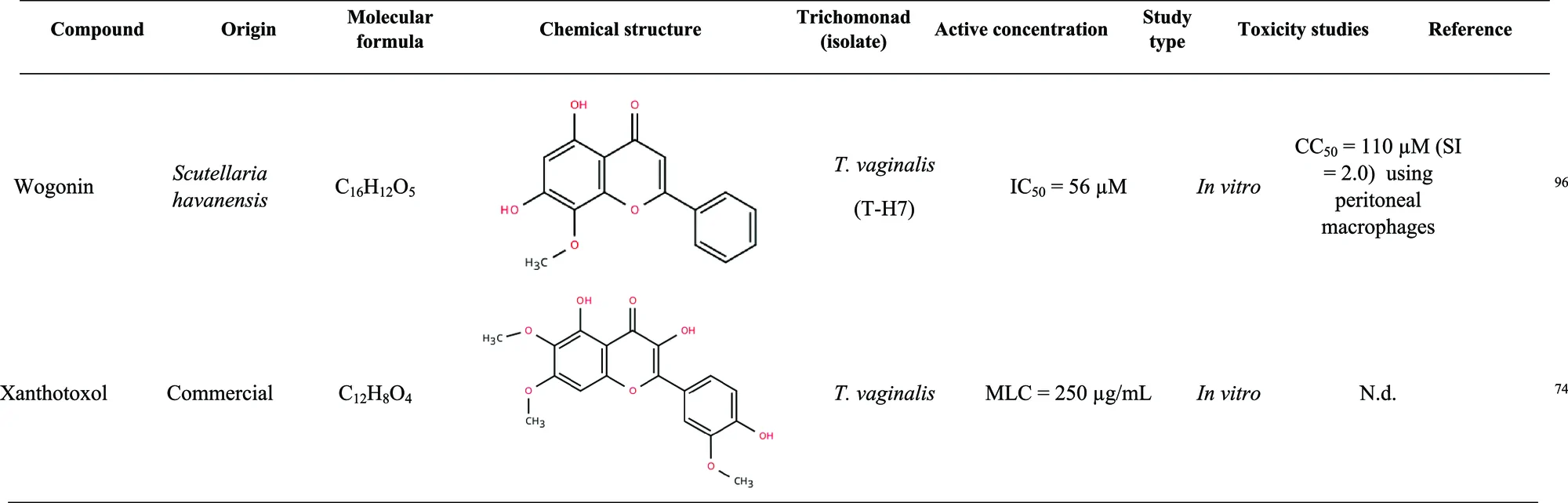
Isolated Natural Compounds with Trichomonacidal Activity Reported against Human and Veterinary Trichomonads^a^

a CC_50_: 50% cytotoxic concentration; HC_50_: 50% hemolytic concentration; IC_50_: half-maximal inhibitory concentration; LC_50_: median lethal concentration; LC_90_: 90% lethal concentration; MIC: minimum inhibitory concentration; MLC: minimum lethal concentration; MTZ: metronidazole; N.d.: not determined; SI: Selectivity Index. ★ indicates selected candidates based on potency combined with at least one additional translational criterion, such as selectivity, activity against resistant isolates, mechanistic evidence, or *in vivo* efficacy.

Among them, the isoquinoline alkaloid berberine, the polyphenol curcumin, and the flavonoid quercetin were comparatively evaluated against *T. vaginalis* (GT15 isolate) by Elizondo-Luévano et al. 2020.^120^ Berberine was the most active compound, showing an IC_50_ of 1.2 μM, whereas curcumin and quercetin were markedly less active, with IC_50_ values of 40.6 and 93.2 μM, respectively. For comparison, MTZ showed an IC_50_ of 0.05 μM under the same experimental conditions. In contrast, antioxidant and cytoprotective effects followed the opposite trend. Quercetin exhibited the strongest antioxidant activity, with an EC_50_ of 1.1 μg/mL, surpassing even vitamin E (EC_50_ = 16.9 μg/mL), while berberine showed the weakest antioxidant effect (EC_50_ = 42.0 μg/mL). Hemolysis assays further indicated that berberine toxicity was concentration-dependent, although concentrations above 2.0 μM remained effective against trophozoites without causing hemolysis. Curcumin and quercetin, in turn, caused less than 2% hemolysis across the tested range and displayed substantially higher cytoprotective activity, with quercetin reaching 89% protection and curcumin 71%, compared with less than 3.5% for berberine.^120^ Taken together, these results suggested that berberine was the most active anti-*T. vaginalis* compound among the three tested molecules, whereas curcumin and especially quercetin may offer better redox balance and membrane protection, highlighting a trade-off between antiparasitic potency and cytoprotective properties among these isolated phytochemicals.

The stronger antiparasitic profile of berberine was reinforced by subsequent studies examining this alkaloid in other plant sources and experimental settings. Berberine was further evaluated as an isolated alkaloid in the study of Elizondo-Luévano et al. 2020,^102^ who investigated methanolic extracts and fractions from the stems and leaves of *Argemone mexicana* against *T. vaginalis* (GT15 isolate). In that work, berberine was identified, together with jatrorrhizine, as one of the major alkaloids present in the most active fractions, and when tested individually, it showed an IC_50_ of 40.7 μg/mL, indicating relevant antitrichomonal activity. This value was lower than that obtained for the crude stem and leaf extracts, supporting the view that alkaloid enrichment increased antiparasitic potency. Given that *A. mexicana* is a medicinal plant widely recognized in traditional medicine and particularly rich in isoquinoline alkaloids, these findings reinforce berberine as one of the main constituents associated with the anti-*T. vaginalis* activity of this species.

A similar trend was observed when berberine and curcumin were reassessed in direct comparison. Pacheco-Ordaz et al. 2021 ^81^ likewise compared the isolated compounds berberine and curcumin against *T. vaginalis* (GT15 isolate) and again found berberine to be the more potent molecule. Berberine showed an IC_50_ of 2.6 μg/mL, whereas curcumin exhibited a markedly weaker effect, with an IC_50_ of 51.11 μg/mL. Under the same conditions, MTZ showed an IC_50_ of 0.07 μg/mL. Despite this difference in antiparasitic potency, both compounds displayed low hemolytic activity, although curcumin was less hemolytic overall, remaining below 2% across the tested concentrations, while berberine showed hemolysis below 8%. These findings reinforce the stronger anti-*T. vaginalis* profile of berberine relative to curcumin, while also showing that potency relative to the reference drug and host-cell compatibility should be considered together when assessing their therapeutic potential.

Within flavonoids, quercetin also emerged as a relevant isolated anti-*T. vaginalis* compound. In this sense, Elizondo-Luévano et al. 2021 ^84^ investigated the activity of quercetin against *T. vaginalis* (GT15 isolate) in parallel with the methanolic leaf extract of *K. daigremontiana*. The flavonoid showed relevant antitrichomonal activity, with an IC_50_ of 21.17 μg/mL, confirming its contribution to the antiparasitic effect previously observed for the crude extract. For comparison, MTZ showed an IC_50_ of 0.09 μg/mL under the same conditions, indicating substantially greater potency. Hemolysis assays further indicated low erythrocyte damage, with approximately 0.7% hemolysis at 1.0 μg/mL.

Wogonin, a major flavone isolated from the chloroform extract of the aerial parts of *S. havanensis*, was also evaluated as an anti-*T. vaginalis* compound. Against the T-H7 isolate, wogonin showed moderate antitrichomonal activity, with an IC_50_ of 56 μM, while cytotoxicity assays in mouse peritoneal macrophages yielded a CC_50_ of 110 μM, resulting in a SI of 2.0. These values were close to those observed for the chloroform extract itself, supporting the view that wogonin is one of the main constituents contributing to the antiparasitic activity of *S. havanensis.* ^96^ However, the relatively low selectivity also suggests that the anti-*T. vaginalis* effect of wogonin may be associated, at least in part, with nonspecific cytotoxicity rather than with a highly selective antiparasitic mechanism.

Coumarin derivatives from *Cnidium monnieri* have also been identified as relevant anti-*T. vaginalis* molecules. Building on previous reports that osthole, xanthotoxol, isopimpinellin, and bergapten are major coumarins in ethanolic extracts of *C. monnieri*,^129–131^ Yu et al. 2022 ^74^ compared their individual effects against *T. vaginalis*. Among the four compounds, osthole and xanthotoxol showed the most pronounced antitrichomonal activity, whereas isopimpinellin and bergapten displayed only weak or negligible inhibition. Kinetic growth assays indicated that osthole completely prevented parasite growth at 500 μg/mL after 48 h, while xanthotoxol produced a comparable effect at 250 μg/mL. In contrast, bergapten did not significantly alter parasite growth, and isopimpinellin caused only subtle inhibition at higher concentrations. Morphological and flow-cytometric analyses further confirmed that osthole and xanthotoxol induced marked trophozoite deformation and destruction, similarly to MTZ, identifying these two coumarins as the principal constituents responsible for the anti-*T. vaginalis* activity of *C. monnieri* ethanolic extracts.

Özel et al. 2024 ^125^ comparatively evaluated cinnamaldehyde, carvacrol, and thymol against two clinical isolates of *T. vaginalis* and one MTZ-resistant reference strain (ATCC50143). Among the three compounds, cinnamaldehyde consistently showed the strongest activity, with MLC values of 31.25 and 7.81 μg/mL for the two clinical isolates and 15.62 μg/mL for the MTZ-resistant strain. Carvacrol showed intermediate activity, whereas thymol generally required higher concentrations to achieve complete parasite killing. For comparison, MTZ displayed MLC values of 24 μM for both clinical isolates and 390 μM for the resistant strain, confirming the reduced susceptibility of ATCC50143. Checkerboard assays further demonstrated synergistic interactions between MTZ and both cinnamaldehyde (FICI = 0.374) and carvacrol (FICI = 0.187), while the combination with thymol showed partial synergy (FICI = 0.750). These findings indicate that, among the three isolated compounds, cinnamaldehyde was the most potent and that both cinnamaldehyde and carvacrol may be particularly valuable as adjuvants to MTZ in resistant *T. vaginalis* infections.

Other authors identified different patterns for the anti-*T. vaginalis* activity of carvacrol and thymol. In the study of Karami et al. 2023,^95^ carvacrol and thymol were the most active isolated compounds, both reaching an MLC of 100 μg/mL after 48 h of incubation, whereas eugenol was less potent, with an MLC of 400 μg/mL. Notably, carvacrol acted more rapidly, already achieving complete parasite killing at 100 μg/mL after 24 h, while thymol required 48 h to reach the same effect. For comparison, MTZ reached MLC values of 12.5 and 6.2 μg/mL after 24 and 48 h, respectively, demonstrating substantially greater lethal activity than the three natural compounds under the same experimental conditions. Consistent with this moderate trichomonacidal profile, Mehri et al. 2020 ^76^ reported that thymol alone displayed an IC_50_ of 85.91 μg/mL after 48 h and produced 91% growth inhibition only at 4000 μg/mL after 24 h, remaining less active than the whole *Trachyspermum ammi* EO from which it derives.

Other plant-derived secondary metabolites have also been examined as isolated anti-*T. vaginalis* compounds. In this context, Gokmen et al. 2019 ^110^ investigated four metabolites isolated from *Haplophyllum myrtifolium*, namely skimmianine, chrysosplenetin, dictamnine, and γ-fagarine. Among them, skimmianine was the only compound to show clear trichomonacidal activity, with MIC of 50 μg/mL after 48 h and MLC of 150 μg/mL. In the same study, MTZ showed a reported MLC of 50 μg/mL. Chrysosplenetin also inhibited parasite motility at concentrations ≥ 50 μg/mL, but no trophozoite lysis was observed, indicating an inhibitory rather than lethal effect. In contrast, dictamnine and γ-fagarine were inactive under the tested conditions. These findings identify skimmianine as the main isolated constituent responsible for the anti-*T. vaginalis* activity of *H. myrtifolium*, while also showing that not all purified metabolites from the same plant contribute equally to the antiparasitic effect.

Particularly noteworthy was lucidin-ω-isopropyl ether, an anthraquinone isolated from *Morinda panamensis*, which showed an IC_50_ of 1.32 μg/mL against *T. vaginalis* (MICH01 isolate), closely approaching the activity of MTZ under the same conditions (IC_50_ = 1.03 μg/mL). Cytotoxicity toward HeLa cells was substantially lower (CC_50_ = 169.23 μg/mL), resulting in a selectivity index of 128.2. This combination of potency and high *in vitro* selectivity distinguishes lucidin-ω-isopropyl ether from many of the other isolated plant compounds evaluated in this review.^127^

Steroidal glycoalkaloids from Solanaceae species have also shown relevant activity against both human and veterinary trichomonads. A comparative study evaluating α-solanine and α-chaconine against *T. foetus* (D1 isolate) and *T. vaginalis* (G3 isolate) showed that α-solanine was the more active compound. At 100 μM, both glycoalkaloids completely inhibited the growth of both parasites, but dose–response analyses showed marked differences in potency; α-solanine exhibited IC_50_ values of 10.86 μM against *T. foetus* and 15.81 μM against *T. vaginalis*, whereas α-chaconine was substantially less active, with IC_50_ values of 35–60 μM against *T. foetus* and *T. vaginalis.* ^121^ These findings indicate that even closely related potato glycoalkaloids can differ markedly in antitrichomonad activity, likely due to structural differences in their trisaccharide moieties rather than in the shared aglycone solanidine scaffold.^122,124^

A still more potent steroidal glycoalkaloid was reported for tomatine, the major tetrasaccharide glycoalkaloid of tomato. Liu et al. 2016 ^119^ showed that tomatine completely inhibited the growth of *T. vaginalis* (G3 isolate) and *T. foetus* (D1 isolate) at 100 μM and displayed IC_50_ values of 7.9 and 1.9 μM, respectively, confirming stronger activity against the bovine parasite. Under the same conditions, MTZ showed IC_50_ values of 0.72 and 0.49 μM against *T. vaginalis* and *T. foetus*, respectively. Nevertheless, the low-micromolar activity of tomatine, particularly against *T. foetus*, distinguishes it among the plant-derived glycoalkaloids evaluated. In contrast, tomatidine, the corresponding aglycone lacking the carbohydrate side chain, showed only weak inhibition in the preliminary screening, supporting the importance of the sugar moiety for antitrichomonal activity. Together, these findings reinforce the role of glycosylation in modulating antiparasitic potency.

Regarding *T. gallinae*, the β-carboline alkaloids harmine and harmaline were individually evaluated following their identification in the alkaloid extract of *P. harmala* seeds. HPLC analysis showed that harmaline and harmine accounted for 3.7 and 1.54% of the total alkaloid extract, respectively, and both compounds exhibited direct *in vitro* activity against trophozoites isolated from pigeons. Harmine was the more active molecule, with a 24 h MIC of 30 μg/mL, whereas harmaline required 100 μg/mL to achieve the same effect. MTZ showed a reported MIC of 50 μg/mL, which suggests greater efficacy for harmine. Notably, both isolated alkaloids were less active than the complete alkaloid extract, which reached a 24 h MIC of 15 μg/mL. This greater activity may reflect additive or synergistic interactions among β-carboline alkaloids or contributions from other minor constituents present in the extract.^69^ However, because formal combination assays were not performed, the existence and molecular basis of such interactions remain to be experimentally demonstrated.

Among the isolated terpenoids evaluated against *T. gallinae*, several monoterpenes showed relevant activity with low toxicity toward mammalian cells. In the study of Bailén et al. 2022,^93^ linalyl acetate, thymol, γ-terpinene, and *p*-cymene were identified among the most active compounds tested individually. Linalyl acetate showed the most favorable profile, with an IC_50_ of 32.2 μg/mL, a CC_50_ above 100 μg/mL in Vero cells, and a SI of 3.1. Thymol also displayed marked activity, with an IC_50_ of 38.4 μg/mL and a SI of 2.6, whereas γ-terpinene and *p*-cymene were slightly less potent, with IC_50_ values of 43.8 and 59.7 μg/mL and SI of 2.3 and 1.7, respectively. For comparison, MTZ showed an IC_50_ of 1.0 μg/mL under the same experimental conditions, indicating substantially greater potency than all four monoterpenes. Since all four compounds showed CC_50_ values above 100 μg/mL, these results indicate that monoterpenes, particularly linalyl acetate and thymol, combine measurable anti-*T. gallinae* activity with relatively low cytotoxicity and may therefore contribute substantially to the activity of the most effective EOs from Lamiaceae species.

Other terpenoids evaluated against *T. gallinae* include β-caryophyllene and α-pinene, identified as the two major constituents of *Psidium guajava* leaf EO. Akbarian et al. 2024 ^101^ reported that β-caryophyllene was the more active compound, causing 95.33% trophozoite mortality at 5 μg/mL after 48 h and yielding LC_50_ and LC_90_ values of 0.32 and 10.31 μg/mL, respectively. Under the same conditions, α-pinene produced lower mortality rates and showed weaker lethal parameters, with LC_50_ and LC_90_ values of 0.73 and 30.50 μg/mL. For comparison, MTZ yielded LC_50_ and LC_90_ values of 2.17 and 9.72 μg/mL, respectively. Although both β-caryophyllene and α-pinene showed lower LC_50_ values than MTZ, β-caryophyllene produced an LC_90_ similar to that of the reference drug, whereas the LC_90_ of α-pinene was substantially higher. Both compounds were therefore more active than the crude EO on the basis of LC_50_ values, but β-caryophyllene emerged as the most active isolated constituent, supporting its role as the main compound associated with the anti-*T. gallinae* effect of *P. guajava* leaf oil.

The combination of natural products with conventional nitroimidazoles may be particularly relevant against drug-resistant parasites because compounds acting through distinct cellular pathways can potentially enhance the antiparasitic efficacy while reducing the concentration required for each agent. For example, the saponin-enriched H100 fraction from *M. rufula* enhanced the inhibitory effect of a sublethal concentration of MTZ against an MTZ-resistant *T. vaginalis* isolate.^112^ Because these saponins induce pronounced parasite membrane damage, their combination with MTZ may produce complementary cellular injury and could potentially facilitate drug access to intracellular targets, although the molecular basis of this interaction has not been directly demonstrated. More recently, checkerboard assays showed synergistic interactions of MTZ with cinnamaldehyde and carvacrol, whereas thymol exhibited partial synergy against an MTZ-resistant *T. vaginalis* strain.^125^ The mechanism underlying these combinations remains unknown, but simultaneous interference with membrane integrity and other cellular processes, together with the reductive activation and macromolecular damage induced by MTZ, represents a plausible multitarget mechanism.

### 2.3 Microbial Natural Products with Trichomonacidal Activity

Microbial natural products have also emerged as a relevant source of bioactive metabolites, expanding the spectrum of antitrichomonal candidates beyond plant-derived compounds (Table 3). In this context, secondary metabolites produced by entomopathogenic bacteria of the genera *Xenorhabdus* and *Photorhabdus* were investigated for antiprotozoal activity using the easyPACId (easy Promoter Activated Compound Identification) approach.^132^ In the initial screening, cell-free supernatants from five *Photorhabdus* and 22 *Xenorhabdus* species were tested against several human protozoan parasites, including *T. vaginalis* (ATCC30001 isolate). Among them, supernatants from *Xenorhabdus* species showed the strongest activity (more than an 80% reduction in parasite viability at 10% of supernatant), whereas none of the tested *Photorhabdus* were active. Promoter exchange mutants further revealed that the antitrichomonal metabolites produced by *Xenorhabdus* were mainly fabclavines, xenocoumacins, xenorhabdins, and PAX peptides. These findings identify bacterial secondary metabolites as an additional and still underexplored source of antitrichomonal compounds beyond plant-derived natural products.^132^

**Table 3:** Microbial-Derived Natural Products with Anti-*T. vaginalis* Activity^a^

origin	organism	extract or product type	trichomonad (isolate)	active concentration	study type	chemical characterization	cytotoxicity	ref
*Amauroderma camerarium*	Fungi	Aqueous extract	*T. vaginalis* (ATCC 30236 and fresh clinical isolates TV-LACH1, TV-LACM2)	MIC (ATCC236) = 2.6 μM; MIC (fresh clinical isolates) = 5.2 μM; MTZ (control)_MIC_ = 8.0, 4.56, and 5.80 μM for ATCC 30236, TV-LACH1, and TV-LACM2, respectively	*In vitro*	Amaurocine	No significant cytotoxicity against human neutrophils (>87% viability after 24 h)	^135^
*Penicillium citrinum* (FMPV 15)	Fungi	Organic extract and its fractions 3A, 4A, and 5A	*T. vaginalis* (ATCC 30236)	Organic extract MIC and IC_50_ = 0.5 and 0.26 mg/mL; Fractions MIC = 1.0 mg/mL	*In vitro*	Citrinin, dicitrinin C, citreoisocoumarin, dihydrocitrinone, decarboxycitrinin, and penicitrinone C	Erythrocytes HC_50_	^136^
							>1.0 mg/mL	
		Citrinin (commercial)	*T. vaginalis* (ATCC 30236*)*	MIC = 200 μM,	*In vitro*	N.d.	Erythrocytes HC_50_	^136^
				IC_50_ = 33.6 μM			>200 μM	
*Penicillium* sp. (KY6863 TV8–3)	Fungi	Pyrrolocin A ★	*T. vaginalis* (G3)	EC_50_ = 0.06 μM	*In vitro*	N.d.	CC_50_ ∼ 21.82 μM	^137^
							(Vero cells); SI = 363	
*Phoma* sp. (CA9310 TV8–3)	Fungi	Phomasetin	*T. vaginalis* (G3)	EC_50_ = 1.8 μM	*In vitro*	N.d.	CC_50_ ∼ 57.78 μM (Vero cells); SI = 32.1	^137^
*Trochila* sp. (BGP15P7IS3)	Fungi	1 (C_24_H_27_NO_5_), 2 (C_25_H_29_NO_5_), 4 (C_25_H_29_NO_4_), 5 (C_24_H_29_NO_4_), 8 (C_20_H_22_O_8_), 9 (isousnic acid), and 10 (usnic acid derivative)	*T. vaginalis* (G3)	*N*-hydroxyapiosporamide and S-isousnic acid reduced parasite viability in ∼ 80% at 25 and 50 μg/mL, respectively;	*In vitro*	N.d.	N.d.	^138^
				MTZ (control)_EC50_ = 3.0 μg/mL				
*Trichocladium* sp. (TN09213 RBM-1)	Fungi	Xanthoquinodin A1	*T. vaginalis* (G3)	EC_50_ = 3.9 μM; MTZ (control)_EC50_ = 3.5 μM	*In vitro*	N.d.	EC_50_ > 25 μM; SI > 6.4	^134^
							(HepG2 liver cells)	
		Xanthoquinodin A2	*T. vaginalis* (G3)	EC_50_ = 6.8 μM	*In vitro*	N.d.	EC_50_ > 25 μM; SI > 3.7	^134^
							(HepG2 liver cells)	
*Xenorhabdus* spp.	Bacteria	PAX peptides, xenorhabdins, xenocoumacins, and fabclavines	*T. vaginalis* (ATCC30001)	*X. cabanillasii, X. doucetiae, X. mauleonii*, *X. hominickii, X. vietnamensis, X. miraniensis*, and *X. kozodoi* showed	*In vitro*	PAX peptides, xenorhabdins, xenocoumacins, and fabclavines	N.d.	^132^
				>80% reduction in parasite viability at 10% of supernatant				

a CC_50_: 50% cytotoxic concentration; EC_50_: half-maximal effective concentration; HC_50_: 50% hemolytic concentration; IC_50_: half-maximal inhibitory concentration; MIC: minimum inhibitory concentration; MLC: minimum lethal concentration; MTZ: metronidazole; N.d.: not determined; SI: selectivity index. ★ indicates selected candidates based on potency combined with at least one additional translational criterion, such as selectivity, activity against resistant isolates, mechanistic evidence, or *in vivo* efficacy.

Fungal metabolites have likewise emerged as relevant sources of anti-*T. vaginalis* agents. Following the earlier observation by King et al. 2019 ^133^ that xanthoquinodins, particularly Xanthoquinodin A1, inhibit *T. vaginalis* growth, Lee et al. 2023 ^134^ expanded the chemical space of this metabolite class by investigating a bioactive extract from *Trichocladium* sp. (isolate TN09213 RBM-1). Their study led to the identification of six previously unreported xanthoquinodin analogues, together with eight known coeluting metabolites. When tested against *T. vaginalis* (G3 isolate), 12 compounds displayed widely variable activity, but the archetypal xanthoquinodins A1 and A2 remained the most effective, with EC_50_ values of 3.9 μM and 6.8 μM, respectively. For comparison, MTZ showed an EC_50_ of 3.5 μM under the same experimental conditions, indicating that xanthoquinodin A1 approached the potency of the reference drug. In contrast, most newly described analogues were less active or inactive at the tested concentrations. Cytotoxicity assays in HepG2 human hepatocytes yielded values above the highest concentration tested (25 μM), corresponding to selectivity indices >6.4 and >3.7 for xanthoquinodins A1 and A2, respectively. These findings indicate a favorable preliminary selectivity profile, particularly for xanthoquinodin A1.

Another recent contribution from fungal natural products came from the endophytic fungus *Trochila* sp. (BGP15P7IS3), isolated from the leaves of *Lilium carniolicum*. In that study, two metabolites, *N*-hydroxyapiosporamide and S-isousnic acid, showed concentration-dependent inhibitory effects against *T. vaginalis* (G3 isolate). *N*-hydroxyapiosporamide emerged as the more active of the two, reducing parasite growth by about 80% at 25 μg/mL, whereas compound S-isousnic acid required 50 μg/mL to produce a comparable effect. MTZ showed an EC_50_ of 3.0 μg/mL in the same study; however, direct comparison of antitrichomonal activity with these metabolites is limited because EC_50_ values were not determined for the natural compounds. These findings further support fungi as a relevant yet still underexplored source of antitrichomonal metabolites and indicate that endophytic species may yield structurally diverse compounds with activity against *T. vaginalis.* ^138^

Proteinaceous fungal metabolites have also shown trichomonacidal activity. Amaurocine, a 12 kDa protein obtained from the aqueous extract of the basidiomycete *Amauroderma camerarium*, demonstrated activity against *T. vaginalis* (ATCC 30236 and fresh clinical isolates).^135^ The purified protein inhibited the ATCC isolate with a MIC of 2.6 μM and was likewise active against two clinical isolates, TV-LACH1 and TV-LACM2, both with MIC values of 5.2 μM. Under the same conditions, MTZ showed MIC values of 8.0, 4.56, and 5.80 μM against ATCC 30236, TV-LACH1, and TV-LACM2, respectively. Thus, amaurocine was more active than MTZ against the reference isolate and showed broadly comparable activity against the two clinical isolates. In addition to its direct antiparasitic effect, amaurocine showed low toxicity toward human neutrophils, maintaining 87.4% viable cells after 24 h of incubation. Interestingly, in the presence of *T. vaginalis*, the protein also increased nitric oxide release by neutrophils, suggesting that its activity may involve not only direct trichomonacidal effects but also modulation of the host inflammatory response.^135^

Further support for fungal metabolites as antiparasitic agents came from the marine-derived fungus *Penicillium citrinum* (FMPV 15). Endres et al. 2022 ^136^ showed that the organic extract of this fungus was active against *T. vaginalis* (ATCC 30236), with IC_50_ and MIC values of 0.26 and 0.5 mg/mL, respectively. Bioguided fractionation on Sephadex LH20 yielded 12 fractions, of which fractions 3A, 4A, and 5A reduced trophozoite viability by 100% at 1 mg/mL. LC-DAD-MS-based metabolomic analysis associated the activity with several polyketide-derived metabolites, particularly citrinin, dicitrinin C, citreoisocoumarin, dihydrocitrinone, decarboxycitrinin, and penicitrinone C. Among these, citrinin was further tested as an isolated compound and showed direct activity against *T. vaginalis*, with an IC_50_ of 33.6 μM and a MIC of 200 μM. Importantly, neither the organic extract nor citrinin caused hemolysis in human erythrocytes. These findings identify citrinin-related metabolites as bioactive fungal-derived scaffolds, although their relatively high effective concentrations and the absence of broader host-cell toxicity and *in vivo* data currently limit their prioritization for further therapeutic development.^136^

Finally, Peramuna et al. 2024 ^137^ identified two highly active fungal tetramic acid derivatives, pyrrolocin A and phomasetin, isolated from *Penicillium* sp. (KY6863 TV8-3) and *Phoma* sp. (CA9310 TV8-3), respectively, against *T. vaginalis*. Among the two compounds, pyrrolocin A was the most active, with an EC_50_ of 0.06 μM, whereas phomasetin showed lower but still relevant activity, with an EC_50_ of 1.8 μM. Cytotoxicity assays in Vero cells yielded CC_50_ values of approximately 21.82 μM for pyrrolocin A and 57.78 μM for phomasetin, corresponding to selectivity indices of approximately 363 and 32.1, respectively. This combination of submicromolar activity and high selectivity particularly distinguishes pyrrolocin A among the microbial natural products identified in this review.

Compared to plant-derived natural products, microbial metabolites may offer some practical advantages for drug discovery and development. Microorganisms can often be cultivated under controlled and reproducible conditions, allowing fermentation parameters to be optimized and potentially facilitating production scale-up while reducing the variability associated with the geographical origin, climate, seasonality, and cultivation conditions of medicinal plants. Moreover, the metabolic diversity of bacteria and fungi provides access to structurally diverse and chemically distinctive scaffolds that may complement those obtained from plant sources. Nevertheless, these advantages do not necessarily translate to greater suitability for therapeutic development. For the microbial natural products evaluated with anti-*T. vaginalis* activity, the available evidence remains predominantly based on *in vitro* assays. Although preliminary cytotoxicity or safety assessments have been reported for some compounds, including xanthoquinodins, amaurocine, citrinin, pyrrolocin A, and phomasetin, further information is still needed regarding their mechanisms of action, pharmacokinetic properties, *in vivo* efficacy, and broader toxicological profiles. Therefore, these metabolites should currently be regarded primarily as bioactive natural products rather than as established drug candidates. Further mechanistic, toxicological, pharmacological, and *in vivo* studies will be essential to determine whether their apparent advantages in production and chemical diversity can be translated into viable therapeutic agents for trichomoniasis.

### 2.4 Nanotechnology-Based Delivery of Natural Products against Trichomoniasis

Nanotechnology represents an alternative approach for drug delivery in the context of trichomoniasis (Table 4). Nanofibers offer flexibility, particularly for intravaginal applications, enabling controlled dosing and meeting specific therapeutic requirements.^139^ In this context, Bellu et al. 2022 ^140^ developed polycaprolactone (PCL)/gelatin nanofibers functionalized with extracts from different parts of *M. communis*, a plant traditionally used in folk medicine for vaginal washing. Phytochemical characterization showed that ellagic acid predominated in fruits and seeds, gallic acid was detected only in leaves, and hydrolyzable tannins represented the major class of phenolics in all extracts, accounting for about 90% of total phenolics in leaves and seeds and 74% in fruits.

**Table 4:** Nanotechnology-Based Formulations of Natural Products Investigated against Trichomoniasis^a^

bioactive compound	delivery system	trichomonad (isolate)	active concentration	study type	main findings	ref
Berberine ★	Nanoparticle	*T. vaginalis* (GT15)	IC_50_ = 0.71 μg/mL	*In vitro*	Encapsulation significantly increased the activity of the products and reduced hemolysis	^81^
*Bunium persicum* EO	Nanoniosomes	*T. vaginalis*	IC_50_ for nanoniosomes containing EO and free EO was 11.01 and 16.09 μg/mL (24 h)	*In vitro*	In HFF cells, formulations exhibited concentration-dependent toxicity; This increase was not significant for formulations containing Tween 60 and 80, and the lowest toxicity was observed for empty niosomes prepared with Tween 80 at 6.25 μg/mL	^141^
*Bunium persicum* EO	Nanoliposomes	*T. vaginalis*	IC_50_ = 45.19 (12 h) and 14.41 μg/mL (24 h)	*In vitro*	The empty liposomal system showed low cytotoxicity toward HFF cells; the cytotoxicity of EO-loaded formulations was not separately reported	^142^
*Capparis spinosa* aqueous extract	Nanoemulsions	*T. vaginalis*	IC_50_ = 99.4 ppm; MTZ (control)_MLC_ = 43.47 ppm (24 h)	*In vitro*	2.8% of hemolysis at 500 ppm	^143^
*Citrullus colocynthis* aqueous extract	Nanoemulsions	*T. vaginalis*	IC_50_ = 99.0 ppm; MTZ (control)_MLC_ = 43.47 ppm (24 h)	*In vitro*	2.3% of hemolysis at 500 ppm	^143^
Curcumin	Nanoparticle	*T. vaginalis* (GT15)	IC_50_ = 4.25 μg/mL	*In vitro*	Encapsulation significantly increased the activity of the products and reduced hemolysis	^81^
*Cymbopogon flexuosus* EO	Nanoemulsion	*T. gallinae*	MLC = 440 μg/mL; MIC = 418 μg/mL; IC_50_ = 110 μg/mL	*In vitro/In vivo*	Treatment with nanoemulsion presented efficiency on the third day p.i.	^105^
*Eucalyptus camaldulensis, Viola odorata*, and *Mentha piperita* (mixture)	Vaginal cream	*T. vaginalis*	EAEX of *E. camaldulensis* at 12.5 mg/mL, AEX of *V. odorata* at 0.3 mg/mL, and HAEX of *M. piperita* at 5 mg/mL showed 100% growth inhibition (24 h). The mixed extract showed 100% growth inhibition	*In vitro*	The prepared vaginal formulation containing stearic acid, triethanolamine, propylene glycol, purified water, and 2.5 mg of *E. camaldulensis*, 0.06 mg of *V. odorata*, and 1 mg of *M. piperita*/1 g of cream; Each gram of cream contained 40.08 μg of 1,8-cineole	^144^
Eugenol	Nanoemulsion	*T. gallinae*	Eugenol and eugenol nanoemulsion MIC = 10 μL/mL (48 h); Eugenol and eugenol nanoemulsion IC_50_ = 4.26 and 1.52 μL/mL (48 h); Treatment of infected pigeons with eugenol nanoemulsions significantly reduced trophozoite numbers, and no motile parasites were detected 4 days p.i.	*In vitro/In vivo*	Eugenol and eugenol nanoemulsion presented CC_50_ = 9.7 (SI = 2.28) and 6.9 μL/mL (SI = 4.54) at 48 h	^145^
*Juglans regia* aqueous extract	AgNPs (Silver nanoparticles)	*T. vaginalis* (ATCC50148)	2 μg/mL inhibited 70% of parasite growth; AgNP-1x and AgNP-2x at 128 and 64 μg/mL caused effective parasite death (24 h); MTZ (control) = 1 up to 128 μg/mL (24 h)	*In vitro*	AgNP-1x and AgNP-2x were the more efficient formulations	^146^
*Micana cordifolia*	Nanoemulsion	*T. vaginalis*	MLC = 1000 ppm	*In vitro*	The formulation was acceptable on the efficacy to eliminate *T. vaginalis*	^147^
*Myrtus communis*	Nanodevices composed of polycaprolactone (PCL) and gelatin nanofibers	*T. vaginalis* (G3)	2NF-S/S and 2NF-S/L formulations = 95% growth inhibition (24 h)	*In vitro*	Both encapsulated and soaked nanofibers exhibited concentration-dependent activity; Transient cytotoxicity in HeLa and no cytotoxic effects were detected in HFF1 fibroblasts or SSC; The nanodevices did not affect the growth of *Lactobacillus acidophilus*	^140^
*Trachyspermum ammi* EO	Nanoliposomes	*T. vaginalis*	IC_50_ = 25.81 (12 h) and 8.08 μg/mL (24 h)	*In vitro*	GC-MS identified thymol (55.87%) as the major constituent; the empty liposomal system showed low cytotoxicity toward HFF cells; cytotoxicity of EO-loaded formulations was not separately reported.	^142^
Thymol	Nanoparticles	*T. vaginalis*	IC_50_ = 10.0, 6.7, and 6.6 μg/mL (24, 48, and 72 h); Thymol nanoparticles IC_50_ = 64.2, 16.4, and 9.55 μg/mL (24, 48, and 72 h)	*In vitro*	The efficacy of the three thymol forms did not significantly differ after 72 h of treatment	^148^
			MTZ (control) = 100% reduction of parasite viability at 65 μg/mL (24, 48, and 72 h)			
	Nanoliposomes	*T. vaginalis*	IC_50_ = 11.8, 7.4, and 4.7 μg/mL (24, 48, and 72 h)	*In vitro*	The efficacy of the three thymol forms did not significantly differ after 72 h of treatment	^148^
			MTZ (control) = 100% reduction of parasite viability at 65 μg/mL (24, 48, and 72 h)			

a AEX: aqueous extract; CC_50_: 50% cytotoxic concentration; EAEX: ethyl acetate extract; EO: essential oil; HAEX: hydroalcoholic extract; HC_50_: 50% hemolytic concentration; HFF: human foreskin fibroblasts; HFF1: human foreskin fibroblast 1; IC_50_: half-maximal inhibitory concentration; LC_50_: median lethal concentration; LC_90_: 90% lethal concentration; MIC: minimum inhibitory concentration; MLC: minimum lethal concentration; MTZ: metronidazole; N.d.: not determined; p.i.: post infection; SI: Selectivity Index; SSC: skin-derived stem cells. ★ indicates selected candidates based on potency combined with at least one additional translational criterion, such as selectivity, activity against resistant isolates, mechanistic evidence, or *in vivo* efficacy.

When incorporated into nanofibers, both soaked and encapsulated preparations inhibited *T. vaginalis* (G3 isolate), with the most effective formulations being 2NF-S/S and 2NF-S/L, corresponding to two soaked nanofiber disks containing seed and leaf extracts, respectively; both reduced parasite viability by 95% after 24 h of incubation. The nanodevices caused only transient cytotoxicity in HeLa cells at early time points, whereas no cytotoxic effects were observed in fibroblasts (HFF1) or skin-derived stem cells at 24 or 48 h. Importantly, the formulations did not interfere with the growth of *Lactobacillus acidophilus*, suggesting the preservation of the beneficial vaginal microbiota and supporting further investigation of myrtle-functionalized nanofibers as a localized delivery platform for the treatment of vaginal infections, including human trichomoniasis.^140^

In addition to polymeric nanofibers, lipid-based nanocarriers have also been explored to improve the delivery and antitrichomonal performance of plant-derived volatile compounds. Siyadatpanah et al. 2023 ^142^ prepared nanoliposomes loaded with EOs from *Bunium persicum* and *Trachyspermum ammi* and evaluated their activity against *T. vaginalis in vitro*. The encapsulation efficiency reached 40% for *B. persicum* and 51% for *T. ammi*. GC-MS analysis showed that the *B. persicum* EO was characterized mainly by caryophyllene (25.51%), whereas thymol (55.87%) predominated in the *T. ammi* oil. Both nanoformulations inhibited parasite growth in a time-dependent manner, with the *B. persicum* liposomes showing IC_50_ values of 45.19 μg/mL after 12 h and 14.41 μg/mL after 24 h, while the *T. ammi* liposomes were more active overall, with corresponding IC_50_ values of 25.81 and 8.08 μg/mL. At 250 μg/mL, both formulations completely suppressed parasite growth after 24 h. The empty liposomal system showed low cytotoxicity toward HFF cells. However, cytotoxicity of the essential oil-loaded nanoliposomes was not separately characterized, limiting conclusions regarding the selectivity of the complete formulations.^142^

A related nanostructured approach was later applied to *Bunium persicum* EO using nanoniosomes, further supporting the value of vesicular systems for improving the antitrichomonal performance of volatile compounds. Moghadam et al. 2024 ^141^ first characterized the oil by GC-MS, identifying γ-terpinene (21.86%) and cuminic aldehyde (17.28%) as the major constituents. Among the tested formulations, the optimized PEGylated niosomal system showed an encapsulation efficiency of 63%, while also providing slow and continuous release under pH and temperature conditions similar to the *T. vaginalis* environment. Nanoencapsulation improved the activity of *B. persicum* EO from an IC_50_ of 16.09 to 11.01 μg/mL after 24 h, while maintaining low toxicity toward HFF cells.^141^

A polymeric nanoparticle strategy was also applied to improve the delivery of plant extracts and isolated phytochemicals with anti-*T. vaginalis* activity. Pacheco-Ordaz et al. 2022 ^81^ encapsulated ethanolic extract of *Curcuma longa* (ExtCl), methanolic extract of *Berberis vulgaris* (ExtBv), and their main constituents curcumin (CurNPs) and berberine (BerNPs) into Eudragit EPO nanoparticles. Encapsulation markedly enhanced activity in all cases. Against *T. vaginalis* (GT15 isolate), the IC_50_ of the *C. longa* extract decreased from 496.36 to 102.29 μg/mL after nanoencapsulation, while that of the *B. vulgaris* extract decreased from 192.08 to 46.30 μg/mL. An even greater improvement was observed for the isolated compounds, with CurNPs and BerNPs reaching IC_50_ values of 4.25 and 0.71 μg/mL, respectively. Hemolysis assays further indicated that both free and nanoencapsulated treatments had low toxicity toward human erythrocytes, and that encapsulation reduced hemolytic activity relative to the nonencapsulated forms. Among these formulations, BerNPs showed the greatest activity (IC_50_ = 0.71 μg/mL), while nanoencapsulation also reduced hemolytic effects relative to the corresponding nonencapsulated treatments.

Other nanostructured formulations have also shown relevant antitrichomonal effects. Al-Ardi et al., 2021,^143^ evaluated nanoemulsions prepared from aqueous fruit extracts of *Citrullus colocynthis* and *Capparis spinosa* against *T. vaginalis* and found a substantial reduction in parasite viability after 72 h of incubation. At 500 ppm, the *C. spinosa* nanoemulsion reduced parasite growth by 87.04%, whereas the *C. colocynthis* formulation reached 89.89% inhibition. Both nanoemulsions showed IC_50_ values close to 100 ppm and low hemolytic activity (<3%), indicating comparable antitrichomonal profiles. MTZ showed a reported MLC of 43.47 ppm in the same study.^143^ Along the same line, Vazini^147^ reported high anti-*T. vaginalis* activity for a nanoemulsion containing *Micana cordifolia* at 1000 ppm, further supporting nanoemulsification as a useful strategy to enhance the biological performance of plant-derived products against trichomonads.

Comparative evaluation of different thymol formulations further illustrated how the type of nanocarrier can influence antitrichomonal performance over time. Jalili et al. 2023 ^148^ assessed pure thymol, thymol-loaded nanoparticles, and thymol-loaded nanoliposomes against *T. vaginalis*. After 24 h of exposure, free thymol remained the most active form, with an IC_50_ of 10 μg/mL, whereas the nanoparticle and nanoliposomal formulations showed IC_50_ values of 64 and 12 μg/mL, respectively. However, these differences diminished with longer incubation. After 72 h, all three preparations displayed comparable activity, with IC_50_ values of 6.6 μg/mL for free thymol, 9.5 μg/mL for thymol-loaded nanoparticles, and 4.7 μg/mL for thymol-loaded nanoliposomes. MTZ, used as the reference drug, completely reduced trophozoite viability at 65 μg/mL at all evaluated time points; however, direct numerical comparison with the thymol formulations is limited because their activities were expressed as IC_50_ values. Taken together, these findings suggest that nanoliposomes preserve the activity of thymol more effectively than polymeric nanoparticles and may represent the more favorable delivery system for this monoterpene.

Metal-based nanostructures have also been tested against trichomonads. Şimşek et al. 2023 ^146^ synthesized silver nanoparticles (AgNPs) using aqueous extract of *Juglans regia* and evaluated their effect on *T. vaginalis* (ATCC50148 isolate). All formulations reduced trophozoite viability by about 70% at 2 μg/mL, indicating substantial activity even at low concentrations. At higher doses, complete inhibition was achieved after 24 h, with the AgNP-1x and AgNP-2x formulations reaching 100% inhibition at 128 and 64 μg/mL, respectively.

Alternative topical formulations have also been proposed as a way to translate plant-derived trichomonacidal activity into locally applicable therapies. In this context, a vaginal cream containing a mixture of extracts from *Mentha piperita*, *Eucalyptus camaldulensis*, and *Viola odorata* was developed. Prior to formulation, the ethyl acetate extract of *E. camaldulensis* leaves (12.5 mg/mL), the aqueous extract of *V. odorata* root (0.3 mg/mL), and the hydroalcoholic extract of *M. piperita* (5 mg/mL) each achieved complete inhibition of *T. vaginalis* viability after 24 h of incubation. Based on these results, the authors selected a combined formulation that also produced 100% trophozoite growth inhibition and incorporated, per gram of cream, 2.5 mg of *E. camaldulensis*, 0.06 mg of *V. odorata*, and 1 mg of *M. piperita* in a base composed of stearic acid, triethanolamine, propylene glycol, and purified water. Gas chromatography further indicated that 1,8-cineole was the major volatile constituent of the final product, with each gram of cream containing 40.08 μg of this compound. These findings illustrate the feasibility of incorporating plant-derived preparations into a locally administered vaginal formulation, although further safety and *in vivo* efficacy studies are required.^144^

By contrast, nanotechnology-based strategies for avian and bovine trichomoniasis remain largely unexplored. No studies were identified for *T. foetus*, and only two reports were found for *T. gallinae*. In one of them, Baccega et al. 2021 ^105^ evaluated a nanoemulsion containing *Cymbopogon flexuosus* EO and reported MIC, MLC, and IC_50_ values of 418, 440, and 110 μg/mL, respectively. Despite this moderate *in vitro* potency, the formulation performed well *in vivo* in Japanese quails (*Coturnix japonica*), becoming effective by the third day of treatment and leading to the complete disappearance of trophozoites from day 4 onward; by day 7, no viable parasites were detected, and no deaths or adverse effects were observed.

A more favorable *in vitro* profile was later reported for eugenol nanoemulsion by Khaki et al. 2024.^145^ After 48 h of incubation, both the nanoemulsion and free eugenol showed the same MIC (10 μg/mL), but the nanoemulsion displayed a lower IC_50_ (1.52 μg/mL) than the isolated compound (4.26 μg/mL). Cytotoxicity assays in Vero cells indicated a CC_50_ of 6.9 μg/mL for the nanoemulsion and 9.7 μg/mL for free eugenol, resulting in an SI of 4.54 and 2.28, respectively. Thus, nanoemulsification improved both the apparent *in vitro* potency and selectivity of eugenol, although the selectivity margin remained moderate. In experimentally infected pigeons, the eugenol nanoemulsion eliminated viable *T. gallinae* by the fourth day of treatment, whereas the same outcome was only observed on the fifth day in the groups treated with free eugenol or MTZ. No mortality occurred during the experiment. Available evidence supports nanoemulsions as valuable platforms for improving the efficacy of plant-derived compounds against avian trichomoniasis.

### 2.5 Trichomonacidal Mechanisms of Action of Natural Products

Numerous natural products have shown activity activity against trichomonads. However, evidence regarding their mechanisms of action remains limited and is concentrated almost exclusively on *T. vaginalis*. No robust mechanistic studies were identified for *T. gallinae* or *T. foetus*. Moreover, the available evidence for *T. vaginalis* is heterogeneous, ranging from ultrastructural observations and biochemical assays to gene-expression analyses and molecular docking studies. Taken together, these data suggest that natural products may interfere with multiple parasite processes, including membrane integrity, virulence-associated proteins, extracellular nucleotide metabolism, redox balance, and enzymes involved in anaerobic energy metabolism (Figure 3).

**Figure 3:**
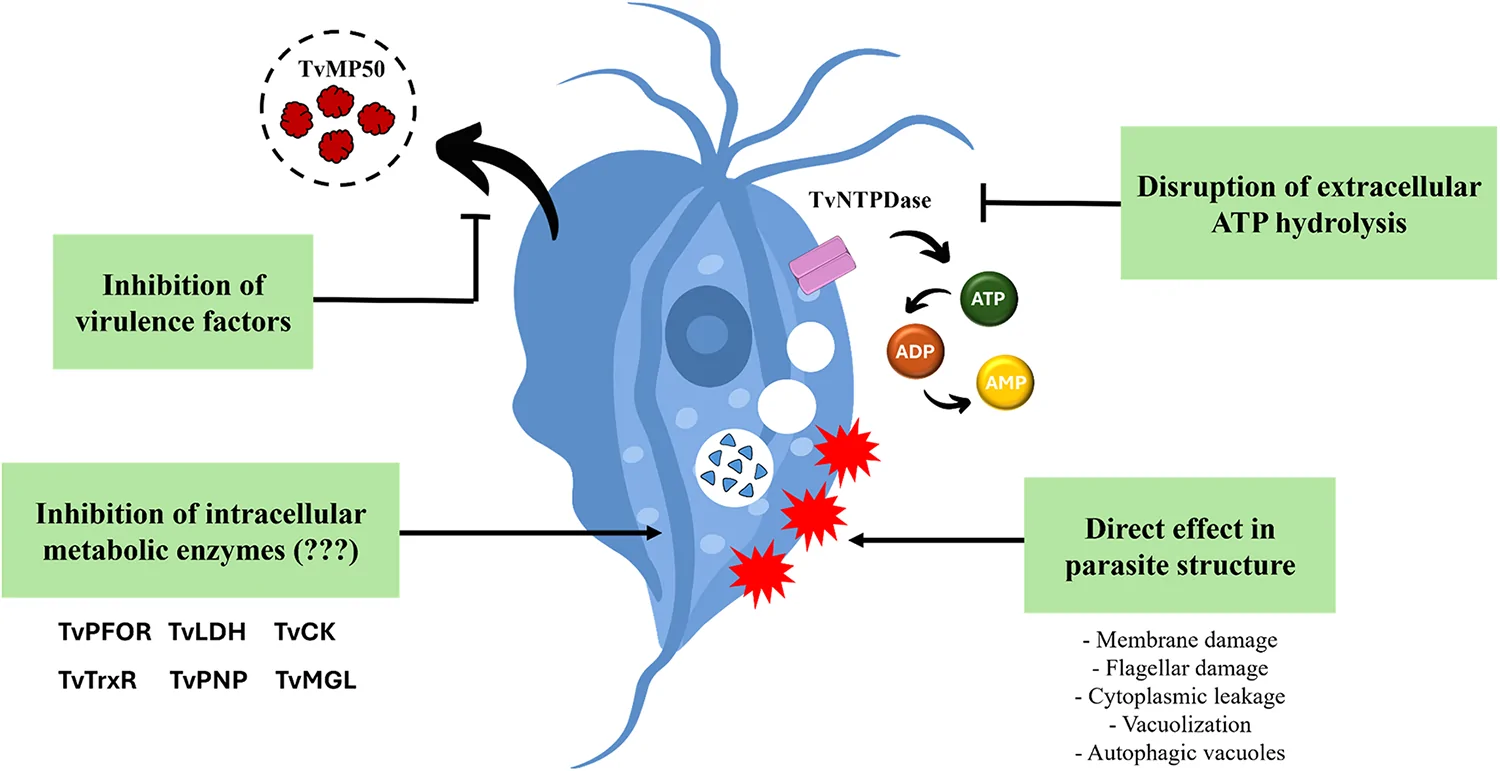
Schematic representation of the proposed antitrichomonal mechanisms of action of natural products identified in this review.

Direct effects on the parasite membrane and organelles are among the most consistently reported trichomonacidal mechanisms described for natural compounds. In this sense, lectins extracted from *P. vulgaris* induced marked ultrastructural damage in *T. vaginalis* clinical isolates after 24 h of incubation, including vacuolization, nuclear destruction, and plasma membrane and flagellar damage, while the axostyle remained preserved.^149^ Lectins are carbohydrate-binding proteins that recognize specific glycoconjugates on biological surfaces^150^ and have already shown antiparasitic effects in other protozoa.^151,152^ Similar structural injury in *T. vaginalis* was described for the monoterpene geraniol, whose exposure led to extensive ultrastructural disorganization, including autophagic vacuoles, dilation of the rough endoplasmic reticulum, chromatin condensation, disappearance of the nuclear envelope, ribosomal loss, organelle disintegration, membrane rupture, and cytoplasmic leakage.^116^ These alterations are consistent with the general mode of action proposed for terpenes, whose marked hydrophobicity facilitates their partition into biological membranes. By interacting with membrane lipids, terpenes may disturb membrane organization and permeability, promote leakage of cytoplasmic contents, and impair the function of membrane-associated and intracellular targets.^153^ In *T. vaginalis*, such effects likely culminate in severe structural collapse and loss of trophozoite viability.^154^

Other studies suggest that natural products may interfere with virulence-associated molecules and extracellular signaling pathways. This is the case of lucidin-ω-isopropyl ether, isolated from *Morinda panamensis*, which showed activity against *T. vaginalis* (MICH01 isolate) and was associated with reduced expression of the *mp50* gene.^127^ TvMP50 is a Zn^2+^-dependent *T. vaginalis* virulence factor that contributes to host-cell interaction and pathogenicity,^155^ making these findings particularly relevant from a therapeutic perspective. Likewise, exposure of *T. vaginalis* (ATCC 30236 isolate) to lycorine, an Amaryllidaceae alkaloid isolated from the bulbs of *Hippeastrum aulicum glaucophyllum*, reduced AMP formation, suggesting inhibition of the nucleoside triphosphate diphosphohydrolase (NTPDase) activity and consequent disruption of extracellular ATP hydrolysis. Pretreatment of parasites with lycorine also induced increased ROS generation by neutrophils.^128^ Because *T. vaginalis* is auxotrophic and lacks *de novo* purine synthesis, depending on adenosine uptake for survival,^156^ NTPDase represents an important enzyme in purine salvage. Its modulation may therefore constitute another plausible pathway through which natural compounds impair trophozoite viability. Additionally, some natural products may enhance the susceptibility of *T. vaginalis* to host immune effector cells, thereby contributing to parasite clearance.

A complementary body of evidence points to enzymes linked to redox regulation and anaerobic metabolism as being relevant molecular targets. Citral (3,7-dimethyl-2,6-octadienal), for example, showed rapid trichomonacidal activity and reduced nitric oxide levels in trophozoites, while docking analyses suggested interactions with thioredoxin reductase (TvTrxR), purine nucleoside phosphorylase (TvPNP), and methionine γ-lyase (TvMGL).^126^ These enzymes are particularly relevant because redox homeostasis and sulfur-containing amino acid metabolism are crucial for survival under low-oxygen conditions in which the microaerophilic parasite *T. vaginalis* thrives.^157,158^ Similarly, betulin and stigmasterol isolated from *Tagetes nelsonii* were predicted to interact with TvPFOR, lactate dehydrogenase (TvLDH), and TvPNP, with stigmasterol showing the strongest binding energies for TvLDH and TvPFOR.^123^ Because pyruvate:ferredoxin oxidoreductase is linked to hydrogenosomal energy metabolism, these *in silico* findings are particularly interesting and suggest that parasite-specific anaerobic enzymes may represent valuable targets for future natural product discovery.

Docking-based studies have also implicated additional enzymes in the modes of action of plant metabolites. *In silico* analysis of compounds identified in the methanolic extract of *Apamarga Kshara* suggested that neophytadiene, methyl eicosanoate, palmitic acid, stearic acid, dodecanol, and related constituents may bind to carbamate kinase (TvCK) with favorable interaction energies.^159^ TvCK is an important enzyme involved in ATP production via arginine metabolism.^160^ The end product of this pathway, ornithine, is readily used in polyamine synthesis, and in trichomonads, this metabolic route is associated with arginine depletion, accompanied by increased putrescine levels in vaginal and seminal fluids.^161^ Although these findings remain preliminary and require biochemical confirmation, they reinforce the possibility that natural products can affect ATP metabolism and other pathways critical for *T. vaginalis* survival. More broadly, they illustrate a recurring pattern in the literature: many natural compounds are proposed to act on enzymes involved in energy generation, purine salvage, and oxidative stress control, but only a minority of studies validate these hypotheses experimentally.

The 20S proteasome has also emerged as a relevant molecular target in *T. vaginalis*. Inhibitors derived from carmaphycin, a marine natural product originally isolated from the cyanobacterium *Symploca* sp., have been demonstrated to selectively target the parasite proteasome.^163^ In particular, the synthetic derivative carmaphycin-17 displayed greater activity against *T. vaginalis* than MTZ, retained activity against MTZ-resistant isolates, and preferentially inhibited the β1 and β5 catalytic subunits of the parasite proteasome compared with their human counterparts.^163^ These findings highlight the 20S proteasome as an additional molecular target that could be explored in studies of plant- and microbial-derived natural products. The antitrichomonal potential of marine-derived natural products has been discussed elsewhere.^164,165^

Whether trichomonads can readily develop resistance to natural products remains largely unexplored since experimental resistance-selection studies are still scarce. Nevertheless, compounds acting through pathways distinct from the reductive activation required by 5-nitroimidazoles may offer an important advantage, particularly against MTZ-resistant isolates. In addition, the ability of some natural products to affect multiple cellular processes or to synergize with MTZ may provide complementary therapeutic strategies and deserves further experimental validation.

Several natural compounds discussed in this Review, including eugenol, thymol, carvacrol, tannic acid, and berberine, also exhibit well-established antibacterial and antifungal activities. In other microorganisms, their effects have been associated with disruption of membrane integrity and ion gradients, impairment of energy metabolism, interference with membrane-associated proteins and efflux systems, and disturbance of redox homeostasis.^166–169^ These observations suggest that some conserved cellular vulnerabilities may contribute to their broad-spectrum antimicrobial activity. However, whether the same mechanisms account for their antitrichomonal effects remains largely unknown and requires direct experimental validation.

Importantly, broad antimicrobial activity does not necessarily imply selectivity toward the parasite or the absence of host toxicity. Differences in membrane composition, metabolic organization, antioxidant capacity, and host absorption and biotransformation may influence selective toxicity, but these effects are strongly concentration- and exposure-dependent. Accordingly, cytotoxicity, SI, bioavailability, and achievable concentrations at the site of infection should be considered together with the antiparasitic potency.^166,170^ Future mechanistic studies should therefore investigate whether membrane disruption, redox imbalance, hydrogenosomal energy metabolism, and intracellular ATP homeostasis contribute to the activity of highly active natural products against trichomonads.

Overall, the mechanistic basis of antitrichomonal activity remains only partially resolved for most of the natural products described so far. This gap is particularly important because trichomonads possess several unusual features and biochemical pathways that could be exploited as novel therapeutic targets. In addition to proteinaceous cytoskeleton structures, including sigmoidal filaments, kinetosomal bodies, rootlet fibers, the costa, and parabasal filaments,^162^ these parasites rely on distinctive anaerobic pathways centered on the hydrogenosome, as well as on tightly regulated thiol-dependent redox metabolism and purine salvage. Current evidence therefore suggests that natural products may affect multiple parasite-specific processes, but a deeper understanding, especially for trichomonads of veterinary importance, is essential to translate these compounds into rational leads.

## 3 Concluding Remarks, Limitations, and Perspectives

Trichomoniasis remains an important yet still neglected challenge in human and animal health, reinforcing the need for new and effective trichomonacidal agents. The evidence compiled in this Review highlights natural products as a chemically diverse and still underexplored source of therapeutic leads. Plant-derived products, microbial metabolites, and natural-product-based formulations have all shown activity against pathogenic trichomonads. Nevertheless, direct comparisons among studies should be interpreted with caution because of substantial differences in the experimental conditions used to define activity. In addition to high antitrichomonal activity, selectivity toward the parasite, reproducibility across isolates, chemical characterization, activity against drug-resistant parasites, mechanistic evidence, and *in vivo* efficacy should also be considered when defining a promising candidate. Importantly, most natural products identified in this Review remain at an early stage of development, with limited information on bioavailability, pharmacokinetics, and *in vivo* safety and efficacy. These gaps currently restrict a robust assessment of their translational potential and should be addressed before therapeutic development can be considered. The major challenges associated with the treatment and therapeutic development of human and veterinary trichomoniasis are comparatively summarized in Figure 4.

**Figure 4:**
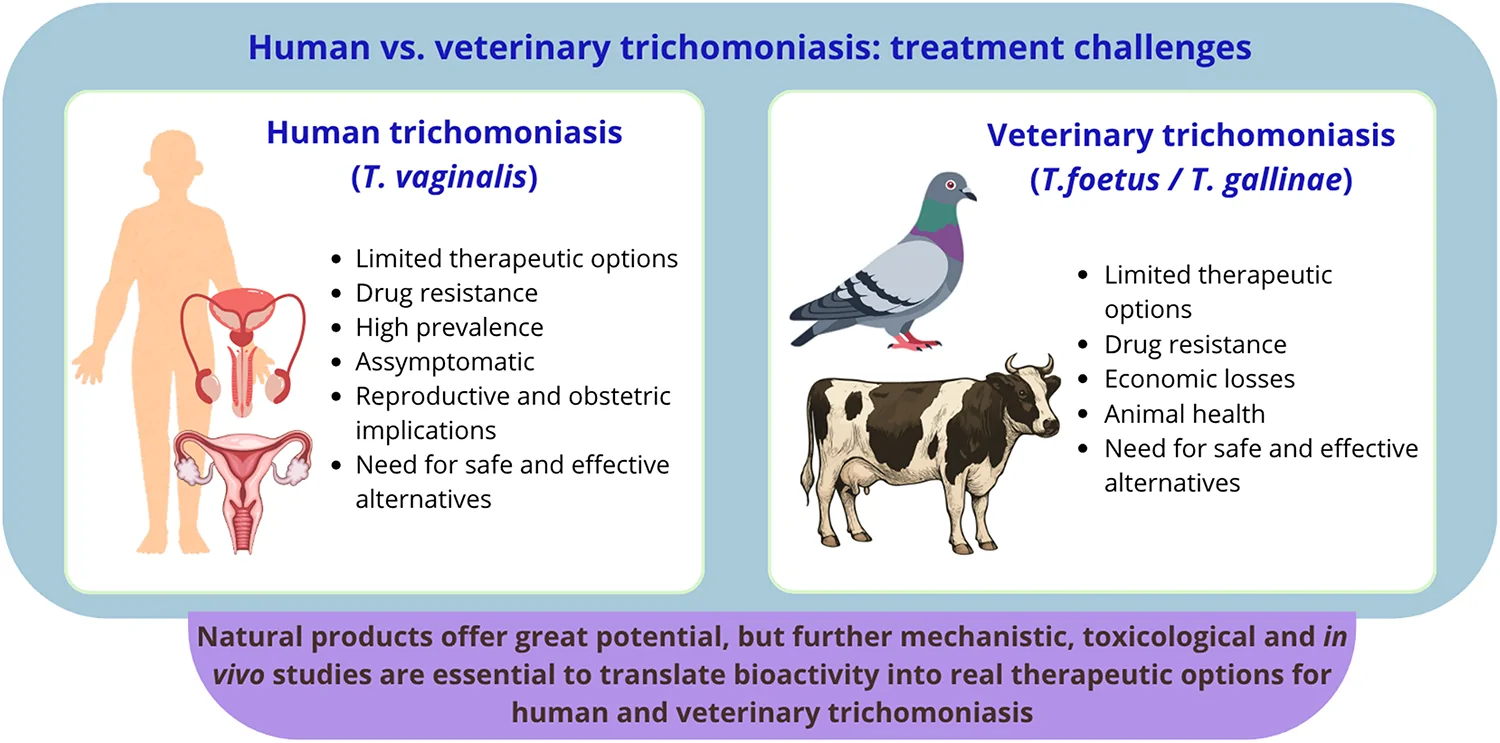
Major challenges in the treatment of human and veterinary trichomoniasis. Comparative overview of the main therapeutic challenges associated with human (*T. vaginalis*) and veterinary (*T. gallinae* and *T. foetus*) infections.

From a One Health perspective, trichomonad infections should also be considered at the interface between human, animal, and environmental health. Although *T. vaginalis*, *T. gallinae*, and *T. foetus* affect distinct hosts and differ in their epidemiological and clinical characteristics, their control faces common challenges, including limited therapeutic options, the emergence or occurrence of drug resistance, and the need for safe and effective alternatives. In veterinary settings, treatment practices may additionally have implications for animal populations, drug exposure, and the surrounding environment, while environmental and ecological factors can influence parasite transmission and host-parasite interactions. The investigation of natural products across human and veterinary trichomonads may therefore provide complementary knowledge for antiparasitic discovery while reinforcing the need to consider therapeutic efficacy, host safety, environmental impact, and responsible drug use as interconnected components of trichomoniasis control.

Despite the substantial heterogeneity among studies, a limited number of natural products emerge as particularly relevant based not only on their antitrichomonal potency but also on additional evidence such as selectivity, activity against drug-resistant isolates, synergistic interactions with reference drugs, or preliminary *in vivo* efficacy. Importantly, the available literature also includes natural products, fractions, and isolated compounds displaying weak or no detectable antitrichomonal activity under tested conditions. For example, extracts prepared from the aerial parts of *P. armeniaca*, *P. pungens* var. *pungens*, *P. sieheana*, and *T. kotschyanus* var. *kotschyanus*, *S. lavandulifolia* subsp. *lavandulifolia*, and *S. verticillata* subsp. *verticillata* were not active against *T. vaginalis*.

However, some candidates identified in this Review deserve particular attention for future investigation. Against *T. vaginalis*, pyrrolocin A displayed remarkable *in vitro* potency and selectivity and would benefit from broader evaluation against clinical and drug-resistant isolates, together with mechanistic and pharmacological studies. Cinnamaldehyde is also noteworthy because of its activity against an MTZ-resistant isolate and its synergic interaction with MTZ. For *T. gallinae*, *A. sieberi* EO and the alkaloid extract of *P. harmala* are particularly relevant because their high *in vitro* activity were subsequently supported by efficacy in experimentally infected pigeons. Both products still need chemical standardization, pharmacokinetic and safety studies, and independent confirmation in larger experimental groups. Evidence for *T. foetus* remains much more limited, but tomatine and *C. nardus* EO represent noteworthy starting points for further investigation, particularly regarding selectivity and *in vivo* proof of concept.

Furthermore, future studies should extend to incorporate computational assessment of drug-like properties and predicted drug metabolism and pharmacokinetic (DMPK) parameters to identify candidates with more favorable profiles for lead optimization. Where structurally related compounds or common molecular targets are available, pharmacophore modeling may further help identify chemical features associated with antitrichomonal activity and guide the development of optimized analogues. Formulation strategies should also be considered at an early stage of therapeutic development, taking into account the site of infection and intended route of administration. For *T. vaginalis*, local intravaginal delivery systems may improve drug exposure while limiting systemic effects, whereas oral formulations may be more appropriate for avian trichomoniasis. For bovine trichomoniasis, formulation development should consider delivery and retention within the reproductive tract, together with animal safety and regulatory requirements for food-producing animals.

An important challenge in natural product research is the standardization of plant-derived extracts and their biological activities. The studies included in this Review originate from different geographical regions worldwide and, consequently, involve plants exposed to distinct climatic and environmental conditions. Factors such as temperature, rainfall, soil composition, light exposure, cultivation practices, and harvesting conditions can influence plant metabolism and favor the production of certain secondary metabolites over others. Consequently, the same plant species collected or cultivated in different geographical regions may exhibit considerable variation in the qualitative and quantitative compositions of their extracts, potentially affecting their biological activity. In addition, differences in plant material, extraction methods, solvents, extraction time and temperature, and subsequent fractionation procedures may further contribute to chemical variability among studies. Therefore, these factors should be carefully considered when comparing the antitrichomonal activity of natural products, highlighting the importance of chemical characterization and standardized extraction protocols to improve the reproducibility and comparability across studies.

One of the major conclusions of this review is the urgent need for greater standardization of *in vitro* antitrichomonal assays. Future studies should preferentially employ quantitative concentration–response experiments rather than measurements at one or a few arbitrarily selected concentrations. As a practical starting framework, crude extracts, fractions, and EOs could be evaluated through 2-fold serial dilutions starting at 500 or 1,000 μg/mL, whereas chemically defined compounds could initially be investigated over approximately 0.1–100 μM, with these ranges extended when justified by preliminary activity, solubility, or physicochemical characteristics. These proposed ranges should not be regarded as formal susceptibility breakpoints, but as a means to improve comparability between screening studies. Whenever possible, EO concentrations should also be reported in mass-based units rather than exclusively as percentages. Dose–response curves should provide IC_50_ or EC_50_ values as the primary quantitative measure of potency, complemented by MIC or MLC values when complete inhibition or parasite killing is evaluated. Since loss of motility does not necessarily demonstrate irreversible parasite death, recovery or regrowth assays after compound removal would further distinguish trichomonastatic from truly trichomonacidal effects.

Assay conditions should also be clearly reported and standardized within each trichomonad species. A 24 h end point, commonly used in susceptibility assays, provides a useful reference for comparison. Experiments should ideally comprise at least three independent biological replicates with appropriate technical replication. At a minimum, untreated parasite, vehicle, and reference drug controls should be included. Vehicle concentrations must be demonstrated to have no independent effect on parasite viability, which is a particularly important consideration for poorly water-soluble compounds and EOs. When possible, candidate compounds should be evaluated against MTZ-sensitive and -resistant *T. vaginalis* and *T. gallinae* isolates, followed by clinical or field isolates as appropriate. *T. foetus* studies should include a well-characterized bovine reference isolate together with field isolates whenever feasible. Selectivity should also become a central criterion in the early trichomonacidal activity discovery pipeline. In this context, the determination of CC_50_ values in host-relevant mammalian or avian cells, followed by calculation of the SI, would help distinguish selective antiprotozoal effects from general cytotoxicity. Moreover, crude extracts, fractions, and EOs should undergo an adequate chemical characterization.

A similar structured progression is required for *in vivo* studies. For *T. vaginalis*, the absence of a universally reproducible vaginal infection model remains a significant barrier to the preclinical development. Recent evidence indicates that a vaginal *T. foetus* infection model can be established more reliably in young mice and may therefore serve as a useful surrogate model for early proof-of-concept studies against *T. vaginalis*,^171^ provided that comparable *in vitro* susceptibility of both species to the candidate has first been demonstrated and that possible differences in molecular targets are considered. For *T. gallinae*, experimentally infected pigeons represent the most biologically relevant model because they are natural hosts and reproduce the infection of interest. For bovine *T. foetus*, estrogenized murine vaginal infection models may provide an accessible first step for efficacy screening. Particularly promising candidates could subsequently advance to the recently established prepubertal heifer model,^172^ which reproduces colonization and inflammatory lesions throughout the bovine reproductive tract while reducing some of the logistical constraints associated with experiments in mature cattle.

The encouraging findings reported to date indicate that some natural products can achieve activity comparable to or even superior to reference treatments under specific experimental conditions. Nevertheless, advancing these candidates toward therapeutic development will require not only efficacy in appropriate preclinical and clinical settings but also adequate chemical standardization, pharmacological characterization, safety assessment, and compliance with the quality requirements expected for therapeutic agents. Mechanistic studies should also remain an integral part of this pipeline since current evidence suggests that natural products may interfere with multiple parasite processes, including membrane integrity, virulence-associated factors, purine salvage, redox homeostasis, and anaerobic energy metabolism. Together with unique trichomonad structures and organelles, these pathways provide promising opportunities for targeted drug development. Importantly, high *in vitro* trichomonacidal activity does not necessarily translate into therapeutic viability, as natural products may fail during subsequent drug development because of unfavorable pharmacokinetic properties, limited bioavailability, insufficient selectivity, toxicity, chemical instability, or difficulties in formulation and standardized production. The major steps and current translational gaps involved in the progression from natural product discovery to potential therapeutic development are summarized in Figure 5.

**Figure 5:**
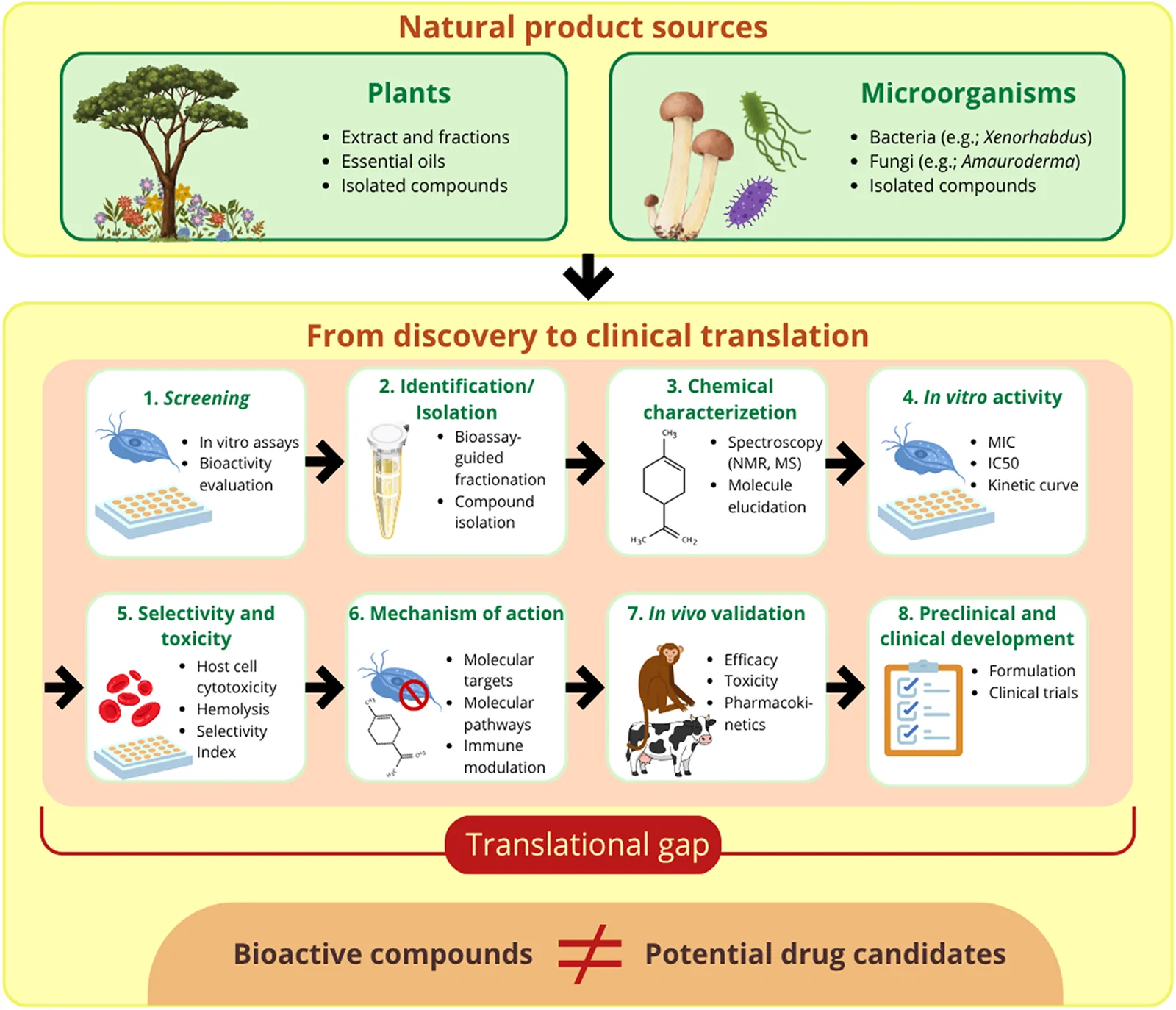
From natural product discovery to clinical translation in trichomoniasis: Schematic representation of the major steps involved in the progression of natural products from initial screening to potential therapeutic development.

In parallel, the future establishment of an accessible repository compiling standardized antitrichomonal bioactivity data could further facilitate cross-study comparisons and guide candidate prioritization. Integrating standardized bioassays, selectivity assessment, mechanistic characterization, and *in vivo* validation will be essential to bridge the “valley of death” and advance natural products from preliminary bioactive leads toward rational therapeutic strategies for human and veterinary trichomoniasis.
